# Unaddressed functional difficulty and care support among White, Black, and Hispanic older adults in the last decade

**DOI:** 10.1093/haschl/qxad041

**Published:** 2023-09-01

**Authors:** Jun Li, Jinkyung Ha, Geoffrey Hoffman

**Affiliations:** Department of Public Administration and International Affairs, Maxwell School of Citizenship and Public Affairs, Syracuse University, Syracuse, NY 13244, United States; Institute of Health Policy and Innovation, University of Michigan, Ann Arbor, MI 48109, United States; School of Nursing, University of Michigan, Ann Arbor, MI 48109, United States

**Keywords:** aging, disparities, long-term care services and supports, health care reform

## Abstract

Unaddressed functional difficulties contribute to disparities in healthy aging. While the Affordable Care Act (ACA) is believed to have reshaped long-term care, little is known on how it has collectively altered the prevalence of older adults with functional difficulties and their use of family and formal care. This study uses nationally representative data from the Health and Retirement Study (2008–2018) to describe racial-ethnic differences in the prevalence of community-dwelling older adults who had difficulty with, but lacked assistance for, self-care, mobility, and household activities before and after the ACA. Individuals with functional difficulties accounted for about one-third of Black and Hispanic individuals, compared to one-fifth of White people. The prevalence of Black and Hispanic people with functional difficulties lacking corresponding care support was consistently 1.5 times higher than that of White people. Racial-ethnic differences disappeared only for low-income households where unaddressed difficulties were uniformly high. While formal care quantity was similar, Black and Hispanic people with functional difficulties received nearly 50% more family care than White people. These gaps between White, Black, and Hispanic older adults were persistent over time. These findings suggest that racial-ethnic gaps in aging needs and supports remain despite major health care reforms in the past decade.

## Lay summary

This study looked at how White, Black, and Hispanic older adults differed in their need for and receipt of help with day-to-day tasks from 2008 through 2018, a period marked by major health care changes spurred by the Affordable Care Act. The study analyzed national data and found that about one-third of Black and Hispanic older adults experienced difficulties with day-to-day tasks, compared to one-fifth of White older adults. The study also revealed that Black and Hispanic individuals with difficulties with day-to-day tasks were 1.5 times more likely to be without care support compared with White older adults. Only low-income populations did not appear to display racial-ethnic differences, where rates of people without care support were high for everyone. Even though more Black and Hispanic older adults had difficulties than White older adults, they were no more likely to receive paid help. Instead, Black and Hispanic older adults relied on help from their family and friends. These differences between groups were consistent over time. The study suggests that, despite efforts to improve health care access in the past decade, there are still significant differences in the care support for older adults from different racial and ethnic backgrounds. More attention and resources are needed to address these disparities and ensure equitable care for all people.

## Teaser

How have gaps in unaddressed functional difficulties and care support between Black and Hispanic and White older adults changed in the past decade in light of major health care reforms? This nationally representative descriptive analysis finds that Black and Hispanic populations consistently had a higher prevalence of people with unaddressed functional difficulties. Moreover, Black and Hispanic older adults continue to lack care support and appear to rely on substantial amounts of family care to manage their higher levels of functional difficulties, despite broad health care policy changes in the recent decade.

## Introduction

Healthy aging,^1^ a process in which functional health is a key driver, is shaped by a broad set of interrelated sociocultural, economic, and health care–related factors.^2-5^ Many older adults with functional difficulties—including challenges with everyday activities like grocery shopping, dressing, or using the toilet—address these long-term care needs and maintain their independence in the community using uncompensated support from family and friends (hereafter referred to as “family care”) or, in some cases, supplemental formal care (paid support for long-term care needs).^6,7^

However, functional difficulties and use of supportive services for racial and ethnic minorities can differ greatly from those of White people, leading to divergent aging experiences in the United States.^8-14^ Although Black or Hispanic older adults are at heightened risk for having functional difficulties, they receive less formal care than White people.^10,11^ Compared with their White counterparts, they have shorter primary care visits,^15^ less annual face time with physicians,^16^ and fewer days in hospice,^17^ while simultaneously experiencing longer hospitalization,^18^ post-acute rehabilitation stays,^19^ and in some contexts, greater use of home- and community-based services.^20^ While the causes of differences in care quantity are multifactorial and contextual,^21^ they are posited to include policy-modifiable factors, such as discrimination, access to care barriers, and other systemic causes.^10,11,22^

The myriad of recent US health policies that are believed to have reshaped long-term care^23^ may have differentially altered the health of community-dwelling older adults, changing the prevalence of people with functional difficulties and how functional needs are addressed across racial-ethnic lines. One potential policy impact is through Medicaid expansion under the 2010 Affordable Care Act (ACA), which was associated with increased access to formal care for low-income adults under 65 years.^24,25^ A recent study found that expansion was associated with an observed 4.4-percentage-point (pp) increase in any long-term care use, and a 3.8-pp increase in home health use.^22^ However, Black and Hispanic individuals may have been especially affected, given lower baseline insurance coverage compared with White people.^26,27^ As a counter example, although ACA's expansion of Medicaid Home and Community-based Services promoted access to community-based formal care broadly,^28^ it may have been more beneficial to White as opposed to Black or Hispanic individuals, given that White older adults are more likely to reside in communities with more formal care supply.^13^

This dynamic of overall, but disparate increases in care use may have been furthered under the ACA's Balanced Incentives Program (BIP), which offered states over $2 billion in enhanced Medicaid matching funds to expand home- and community-based care. While the policy was associated with a 3% increase in daily caregiving in BIP-adopting states, likely due to shifts from nursing to community care, it disproportionately benefited caregivers with higher incomes.^29^ This disparity may result from more challenges for lower-income caregivers associated with social determinants of health (eg, housing instability, health literacy, geographic isolation).^29^

Beyond Medicaid expansion, several of Medicare's alternative payment programs may have also impacted long-term care use. For instance, both of the Bundled Payments for Care Improvement (BPCI) Initiative and Medicare Shared Savings Program (MSSP) have reduced costly post–acute care use. The BPCI was associated with 0.4% and 0.7% reductions in skilled nursing facilities (SNFs) and inpatient rehabilitation facility care and a 0.2% increase in home health agency services.^30^ The MSSP has been associated with fewer discharges to facilities rather than home and shorter SNF stays and home health episodes.^31,32^

Given the substitutability of formal and family care, particularly for lower-income individuals, changes in post–acute care use could shift the distribution of formal and family care use.^33-35^ For instance, Golberstein et al^34^ found that a 1-unit relative decrease in the use of home health services results in a more than a half-unit relative increase in informal care hours; this effect was most pronounced among lower-income families, who may not have adequate resources to privately purchase formal home care and therefore are likelier to replace costlier care with more family care hours. Moreover, if program participants avoid higher-risk, and costlier patients, this could have implications for differences in needs and care by race and ethnicity. Yet, few studies have examined the evolution of long-term care demands and use among older adults, and, in particular, formal and family care patterns in the context of these broad policy shifts.^7^

In particular, how large-scale, and widely varying, policy changes with potentially opposing effects have collectively affected persons of White, Black, and Hispanic racial-ethnic backgrounds is unknown. Coe and Werner,^36^ for instance, examined the prevalence of people with unaddressed functional difficulties among community-dwelling older adults in 2016 but did not examine changes over time or by race or ethnicity. Van Houtven and colleagues^7^ examined care support receipt trends from 2004 to 2016 between White, Black, and Hispanic individuals who were 65 or older with multiple functional difficulties. However, these findings elide potential policy effects on younger, older adults that are expected given policies like Medicaid expansion. Moreover, little is known about shifts on the quantity of care support for functional difficulties—an important dimension for potential inequities.

We aimed to provide a nationally representative picture of unaddressed functional difficulties and corresponding care support among White, Black, and Hispanic community-dwelling older adults in the United States from 2008 through 2018. We used 6 waves of data from the longitudinal Health and Retirement Study (HRS). To proxy for functional difficulties, we used respondents’ reported need for help with activities of daily living (ADLs) or instrumental ADLs (IADLs). A lack of care support for a specific functional difficulty was based on respondent report of no receipt of either formal or family care for their corresponding functional difficulty (eg, reported no help with using the toilet if they indicated difficulty with this activity). Next, we measured the weekly overall quantity of hours of care received by people with any functional difficulty, summed across all functional difficulties. For each population, we estimated the prevalence (and changes in prevalence) of (1) people with any functional difficulties, (2) people with functional difficulties lacking corresponding care support, and (3) weekly hours of family and formal care received by people with any functional difficulties. To explore potential impacts of access to insurance on disparities, namely Medicaid before and after expansion and Medicare, we reconducted analyses for populations with incomes below and above eligibility thresholds to the Medicaid program and for populations under and over age 65 years.

## Data and methods

### Data and variables

We used the RAND HRS Longitudinal File,^37^ a cleaned version of HRS's core data, to examine HRS waves 2008 through 2018. The HRS is a national, biennial panel survey of Americans over the age of 50 and their households that includes data on sociodemographic characteristics, functional difficulties, and help received for each functional difficulty.^38^

Our sample consisted of community-dwelling individuals (ie, not in a nursing home) aged 55 years or older in waves 2008 through 2018 of the HRS (98 004 person-waves) who were non-Hispanic White (hereafter, “White”; 63 923 person-waves), non-Hispanic Black (hereafter, “Black; 17 946 person-waves), or Hispanic (7701 person-waves) (Appendix Table S1). We restricted the sample to individuals ages 55 years or older because the HRS refreshes its survey population with cohorts ages 51 and older every 6 years, with only adults 55 years or older consistently included in each wave.

#### Prevalence of people with any functional difficulties

We considered people as having any functional difficulty if they (or a proxy reported them as) having any difficulty with at least 1 of 11 activities due to health or memory problems, or if they did not or could not do at least 1 of those activities.^7^ The recall period was the last 2 years or since the last survey wave. These activities were (1) 6 ADLs representing self-care activities and mobility (eating, dressing, bathing, walking, getting into or out of bed, and using the toilet) and (2) 5 IADLs representing household activities (meal preparation, grocery shopping, making phone calls, managing money, and managing medications).

#### Prevalence of people lacking care support for functional difficulties

We categorized a person as someone lacking care support if a respondent or proxy reported at least 1 ADL or IADL difficulty (ie, an individual with any functional difficulty) for which no corresponding assistance was received during the past month; assistance was evaluated as support from a family or formal caregiver or through the use of relevant equipment (ie, device for walking or equipment to help with getting in or out of bed), following approaches taken in prior literature.^36^ Given the substantial needs among community-dwelling individuals who can perform tasks with some difficulty, we chose to identify difficulties that were unsupported rather than a failure to complete a specific functional task (which narrowly reflect unmet need). The former offers a broader portrait of community need than the latter and should be interpreted accordingly.

#### Average quantity of care per person per week

We calculated average weekly care hours summed across all 11 ADL or IADL activities, by formal and family care type, that individuals with any functional difficulty reported having received during the past month. Formal care hours included care from an organization, an “institution” employee, a paid helper, or a health care professional. Family care hours included uncompensated care from family (eg, spouse/partner, child, in-laws) and friends.

### Analysis

We compared the risk-adjusted prevalence in people with functional difficulties and corresponding care support of White, Black, and Hispanic people in the past decade. To avoid discounting differences in outcomes that result from cumulative race- and ethnicity-related disadvantages, including physical health and access to and use of health care,^39,40^ we limited risk adjustment to age, age squared, sex, marital status, and children. For each descriptive statistic, we estimated 95% CIs adjusted for survey weighting.

In pooled analyses, we combined all survey waves to estimate the following for White, Black, and Hispanic individuals: (1) prevalence of people with any functional difficulties, (2) prevalence of people with functional difficulties lacking corresponding care support, and (3) average weekly hours of each of family and formal care. The prevalence for a specific group was calculated by dividing, within each population, the number of people with the outcome (eg, any functional difficulty) by the total sample size. For prevalence estimates (1) and (2), we included data from all respondents. For weekly hours of care, (3), we included data only from respondents with at least 1 functional difficulty. To evaluate insurance coverage differences that could explain care support disparities, we calculated prevalence rates by poverty (≤138% of the Federal Poverty Level [FPL] or not) and age (≥65 y or not).^41^ We chose 138% of the FPL because the ACA extended Medicaid eligibility to adults with incomes up to an effective FPL threshold of 138%^42^; we chose 65 years as a cutoff because Medicare, which impacts access to insurance and health care services, primarily enrolls Americans over age 65.

In cross-sectional trend analyses, we used repeated cross-sections instead of pooled data. Trend analyses were otherwise identical to pooled analyses. For further clarity, we showed main results separately for ADL and IADLs.

### Robustness checks

First, given ambiguities in the characterization of functional limitations and care support, we adopted an alternative method to categorize whether a person lacked care support. This approach allows that individuals may report functional difficulty even with the use of assistive equipment (ie, equipment does not eliminate difficulty with walking or getting in or out of bed).^43^ This alternative approach yields a higher prevalence of people lacking care support but similar between-group patterns (Appendix Figure S8).

Second, we examined trends in the prevalence of people with functional difficulties receiving any (regardless of amount of) care support as a way to situate our findings within the existing literature. We found results consistent with findings by Van Houtven and colleagues,^7^ suggesting no differences in the receipt of any care between White and Black and Hispanic individuals. These results demonstrate that binary measures (care receipt or not) can belie important divergences in care support (Appendix Figure S9).

Third, we examined trends in the number of functional difficulties among people with any difficulties. We used the number of functional difficulties as a proxy for disability level.^44^ Changes in disability levels over time were not statistically significant, but patterns suggest potential decreased severity in the period after the ACA (Appendix Figure S10).

## Results

**Figure 1.**
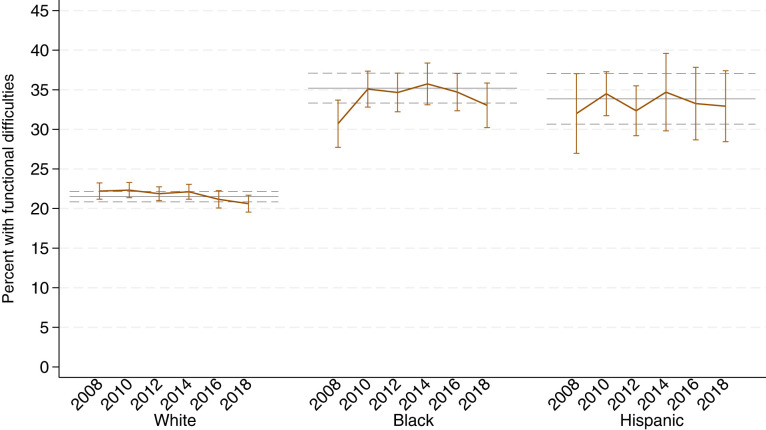
Percentage of people who had functional difficulties, Health and Retirement Study (HRS) waves 2008–2018. Source: Authors’ calculations using data from the 2008–2018 waves of the HRS for community-dwelling individuals at least 55 years of age. All statistics were weighted to account for sampling and to make them nationally representative. Annual estimates and associated 95% CIs were derived from HRS cross-sections and adjusted for sex, age, marital status, and children. Averages across all waves and associated 95% CIs were derived from pooled cross-sections. Functional difficulties included activities of daily living (ADLs) = eating, dressing, bathing, walking, getting into or out of bed, and using the toilet;and instrumental ADLs (IADLs) = meal preparation, grocery shopping, making phone calls, managing money, and managing medications. A person had 1 or more functional difficulties if they or a proxy reported them as having difficulty with the activity due to health or memory problems since the last survey wave or in the last 2 years. White (63 923 person-waves) = non-Hispanic White. Black (17 946 person-waves) = non-Hispanic Black. Hispanic (7701 person-waves).

From 2008 to 2018, the prevalence of older people with any functional difficulties was higher for Black and Hispanic than White populations. Pooled estimates show that 35.2% (95% CI: 33.3–37.1%) of Black and 33.9% (95% CI: 30.7–37.1%) of Hispanic compared to 21.5% (95% CI: 20.8–22.2%) of White people had at least 1 functional difficulty (Figure 1). There was little evidence that these between-group differences diminished over time. The prevalence of Black and Hispanic people with any functional difficulties was over 30% in each of the survey waves, which was consistently 1.5 times greater than the corresponding prevalence among White people.

**Figure 2.**
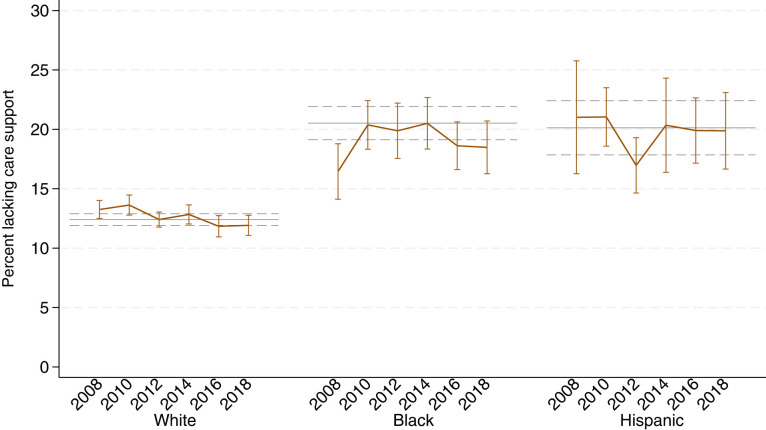
Percentage of people with unaddressed functional difficulties, Health and Retirement Study (HRS) waves 2008–2018. Source: Authors’ calculations using data from the 2008–2018 waves of the HRS for community-dwelling individuals at least 55 years of age. All statistics were weighted to account for sampling and to make them nationally representative. Annual estimates and associated 95% CIs were derived from HRS cross-sections and adjusted for sex, age, marital status, and children. Averages across all waves and associated 95% CIs were derived from pooled cross-sections. Activities of daily living (ADLs) = eating, dressing, bathing, walking, getting into or out of bed, and using the toilet. Instrumental ADLs (IADLs) = meal preparation, grocery shopping, making phone calls, managing money, and managing medications. A person was considered to have lacked care support if they or a proxy reported them as having difficulty with the activity due to health or memory problems but did not receive assistance from a family or formal caregiver or through the use of relevant equipment during the last month. White (63 923 person-waves) = non-Hispanic White. Black (17 946 person-waves) = non-Hispanic Black. Hispanic (7701 person-waves).

Similar to functional difficulties, there were between-group differences in corresponding care support (eg, difficulty with but no help received for toileting), with 20.5% (95% CI: 19.1–21.9%) of Black and 20.1% (95% CI: 17.8–22.4%) of Hispanic compared to 12.4% (95% CI: 11.9–12.9%) of White people who lacked corresponding care support for at least 1 reported functional difficulty. Again, these differences across groups were persistent over time (Figure 2).

**Figure 3.**
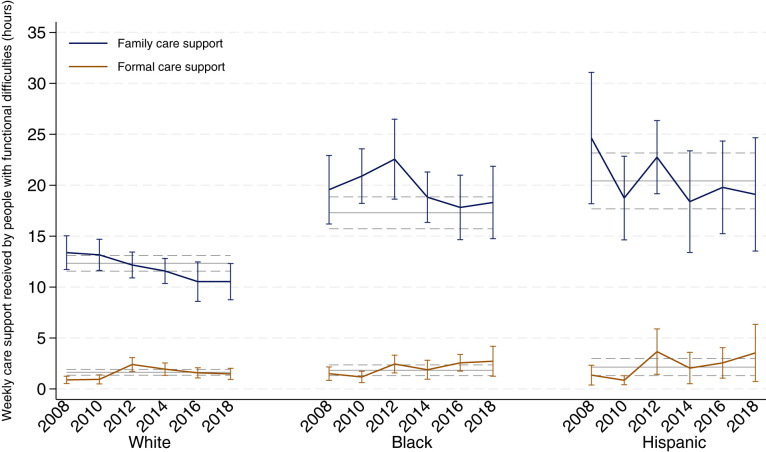
Average weekly hours of family and formal care received by people with any functional difficulties, Health and Retirement Study (HRS) waves 2008–2018. Source: Authors’ calculations using data from the 2008–2018 waves of the HRS for community-dwelling individuals at least 55 years of age. All statistics were weighted to account for sampling and to make them nationally representative. Annual estimates and associated 95% CIs were derived from HRS cross-sections and adjusted for sex, age, marital status, and children. Averages across all waves and associated 95% CIs were derived from pooled cross-sections. All hours were based on respondent (or proxy) report of care support received in the last month. Family care hours included hours of uncompensated care from family (eg, spouse/partner) and friends. Formal care hours included care received from an organization, an “institution” employee, a paid helper, or a health care professional. White (15 951 person-waves) = non-Hispanic White. Black (6174 person-waves) = non-Hispanic Black. Hispanic (2658 person-waves).

Next, focusing on people with any functional difficulties, we examined the quantities of family and formal care support, overall (pooled across all waves) and across waves. While all groups received substantial family care amounts, White people received an average of 12.3 (95% CI: 11.6–13.1) compared to 17.3 (95% CI: 15.7–18.9) weekly family care hours among Black people and 20.4 (95% CI: 17.7–23.2) among Hispanic people. In contrast, average weekly formal care hours were statistically indistinguishable, at 1.6 (95% CI: 1.3–1.9) hours for White, 1.8 (95% CI: 1.3–2.4) hours for Black, and 2.1 (95% CI: 1.4–2.8) hours for Hispanic people (Figure 3).

Trend analysis results suggest, for all groups, respective decreases and increases in family and formal care, although these estimates were modest, imprecise, and did not indicate strong patterns or decreases in gaps between groups (Figure 3).

**Figure 4.**
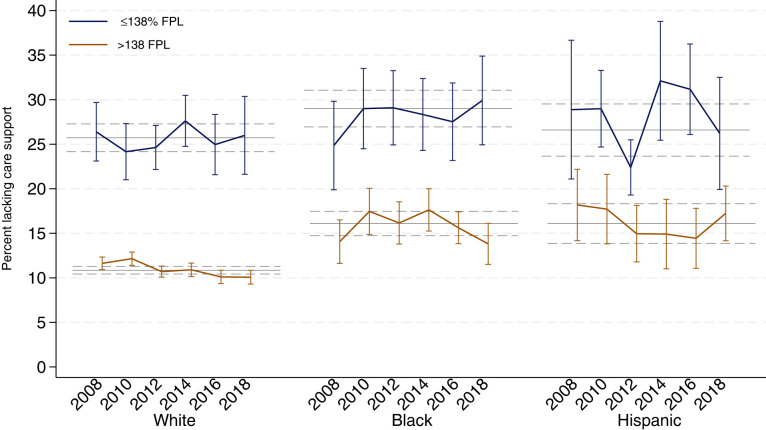
Percentage of people with unaddressed functional difficulties, by household-income-to-poverty ratio, Health and Retirement Study (HRS) waves 2008–2018. Source: Authors’ calculations using data from the 2008–2018 waves of the HRS for community-dwelling individuals at least 55 years of age. All statistics were weighted to account for sampling and to make them nationally representative. Annual estimates and associated 95% CIs were derived from HRS cross-sections and adjusted for sex, age, marital status, and children. Averages across all waves and associated 95% CIs were derived from pooled cross-sections. Activities of daily living (ADLs) = eating, dressing, bathing, walking, getting into or out of bed, and using the toilet. Instrumental ADLs (IADLs) = meal preparation, grocery shopping, making phone calls, managing money, and managing medications. A person was considered to have lacked care support if they or a proxy reported them as having difficulty with the activity due to health or memory problems but did not receive assistance from a family or formal caregiver or through the use of relevant equipment during the last month. White (63 923 person-waves) = non-Hispanic White. Black (17 946 person-waves) = non-Hispanic Black. Hispanic (7701 person-waves). Abbreviation: FPL, Federal Poverty Level.

When examined by income (Figure 4) and age (Appendix Figures S2–S5), patterns were generally similar to our main approach except for people in poverty. The prevalence of lower-income people lacking care support was consistently high over time, and higher than for those aged 65 and older (Appendix Figure S5), but rates were statistically indistinguishable between White, Black, and Hispanic populations. Conversely, the overall prevalence was lower among higher-income individuals, but disparities were observed, with higher rates for Blacks and for Hispanics (Figure 4).

## Discussion

In this nationally representative study, we found that, throughout the period from 2008 to 2018, the prevalence of Black and Hispanic older people with functional difficulties and lacking corresponding care support was consistently around 50% higher than that of White people. More than 30% of older Black and Hispanic compared to 20% of older White people had 1 or more difficulties with functional tasks involving self-care, mobility, or household activities. One-fifth of Black or Hispanic compared to just over one-tenth of White older adults lacked corresponding care support. These gaps persisted despite 40–65% greater amounts of family care and similar amounts of formal care among Black and Hispanic compared to White people. For lower-income individuals, the prevalence of unaddressed functional tasks was consistently high across all racial-ethnic subgroups. Our findings imply that, collectively, recent US health care reforms were not associated with reductions in racial-ethnic gaps in functional health and care needs.

Prior analyses have documented large differences in functional support across racial-ethnic groups. For instance, 15% and 21% of Black and Hispanic compared to 9% of White individuals ages 70 years and older have unaddressed self-care or mobility needs.^45^ Edwards and colleagues^43^ estimated a 22% higher prevalence of people lacking care support among Black compared to White older women with cognitive impairment during the period 2000–2014. Our investigation confirms these racial-ethnic differences as well as their persistence into the period after the ACA's implementation. Together, these findings suggest that the gaps were resistant to broad policy changes, even though clinical and long-term care expansion under Medicaid and novel payment systems in Medicare might be expected to predominantly affect those with the least access to high-quality clinical and supportive services, with no evidence of gains over time for lower-income populations eligible for Medicaid expansion.

Together, these findings suggest that the gaps were resistant to broad policy changes, even though clinical and long-term care expansion under Medicaid and novel payment systems in Medicare might be expected to predominantly affect those with the least access to high-quality clinical and supportive services. Cross-pressures of the programs may have had a cancelling effect. For instance, Medicaid expansion could have increased formal home health care while potentially lowering the use of family care, even as the BIP shifted care back from formal to family care. The findings also reveal persistent unaddressed difficulties among lower-income individuals that surpass those of older age groups, regardless of race; this suggests critical needs in the future that were not addressed despite widespread incentives for improved care access in Medicaid.

Despite the greater prevalence of people with functional difficulties and more difficulties per person, hours of formal care received by Black and Hispanic older adults only modestly (and not statistically significantly) increased in the post-ACA period. These small changes in formal care receipt are in line with other literature showing an approximately 4-pp increase in the likelihood of any formal long-term care use among older adults following Medicaid expansion.^7,24^ While our study was not designed to assess causal implications of Medicaid expansion or other policy changes, our descriptive findings showed no evidence of shifts across groups in the prevalence of people lacking care support among low-income households from before to after the ACA, despite marginal increases in formal care. Thus, to close racial-ethnic gaps, more concerted and broadly targeted policy efforts to direct and tailor formal care equitably are still needed.

Family care, typically outside the scope of health care reform, remained the dominant care type among Black and Hispanic people with functional difficulties. The higher levels of family care received by Black and Hispanic relative to White older adults could be due to differing cultures of family and formal caregiving in the presence of functional needs.^9,10,46,47^ As an example, the notion that family well-being is more important than that of the individual among some Hispanic caregivers has been posited as 1 reason for the low use of formal home care.^48,49^ This heavy reliance on family care could also reflect continued struggles faced by racial-ethnic minorities in accessing high-quality formal care. For instance, the 2022 National Healthcare Quality and Disparities Report shows that Black and Hispanic people had worse outcomes than White people for the majority of access-to-care measures.^50^ Moreover, racial-ethnic minorities tend to be overrepresented in low-quality nursing homes and home health agencies,^51,52^ which may decrease the appeal of formal over family care.^10^ The more limited use of nursing home care may further explain the persistently higher prevalence of functional need among community-dwelling racial-ethnic minorities, and resulting equity issues in unaddressed needs. Other potential factors explaining discrepancies in family care use are the “opportunity costs,” which are higher for higher- versus lower-income families, or greater care needs due to social determinants of health (costs related to housing instability, transportation, home repair needs, and lack of supply of formal care providers due to geographic isolation or other factors).^29^

How such significant reliance on family care among racial-ethnic minorities affects disparities in care support is unclear. On one hand, extensive use of family care among racial-ethnic minorities may reduce the prevalence of people lacking care support, potentially mitigating inequities. On the other hand, family care may be an imperfect substitute for formal care, especially as severity of needs increases.^53^ Alternative payment models that disincentivize the use of institutional post-acute and other formal care are increasingly prevalent,^54,55^ potentially increasing the prevalence of community-dwelling people with functional difficulties and raising pressures on family care.^56^ For racial-ethnic minorities who are already particularly more likely to have lower household and community resources than White people, these systemic changes exacerbate concerns about the adequacy of family care in meeting growing care support needs.^57^ Finally, heavy reliance on family care by racial-ethnic minorities may introduce inequities for caregivers in the future,^58^ since caregiving often comes with mental, physical, and financial hardships.^59-61^ Future work should examine how formal and family care can be leveraged to decrease population-level differences in functional difficulty, paying particular attention to how needs are being addressed across disparate groups.

### Limitations

Our study has several limitations. First, we used repeated cross-sections in trend analyses without adjusting for multiple observations per person. While consistent with approaches in prior literature,^7^ it may not represent the true degree of statistical uncertainty.

Second, while the HRS collects race and ethnicity data, only Black and Hispanic individuals are oversampled, leading to small samples for other racial groups. Therefore, by focusing on groups with larger samples, our study does not capture experiences of other populations, limiting the policy implications of our findings.

Third, the 2018 HRS had skip-pattern issues that prevented some respondents from being asked ADL questions.^37^ Although the HRS imputed the missing data, there may still be missing data bias.^62^

Fourth, our measure of unaddressed functional limitations does not capture unmet need for assistance with ADL/IADLs. The HRS does not ask respondents whether they were unable to complete a task due to lack of assistance or whether, despite receiving help, they remain unable to complete the task. For this reason, our estimates may overstate unmet need (defined as inability to complete tasks) and instead offer broader evidence of incompletely addressed needs. However, any resultant bias is likely consistent over time and should not vary by race and ethnicity.

## Conclusion

Even with large-scale policy changes brought forth by the ACA, this descriptive analysis provided evidence that Black and Hispanic older adults living in the community were still more likely than their White counterparts to experience functional difficulty and lack care support. Lower-income individuals, in particular, showed evidence of substantial needs unaddressed by caregivers. Despite a greater prevalence of people with difficulties as well as more difficulties, the quantity of formal care used by Black and Hispanic older adults did not meaningfully increase relative to White people in a period that included multiple health care reforms that could impact long-term care use. Our descriptive analyses should encourage policymakers and health groups to systematically identify, understand, and address policy-modifiable disparities.

## Supplementary Material

qxad041_Supplementary_Data
