# Supplementary material for: Unaddressed functional difficulty and care support among White, Black, and Hispanic older adults in the last decade
Source: Health Aff Sch. 2023 Sep 1;1(3):qxad041. doi: 10.1093/haschl/qxad041 (PMC10809881; doi:10.1093/haschl/qxad041)
Supplement: qxad041_Supplementary_Data [file qxad041_Supplementary_Data.zip › SupplementalMaterialRR3.docx]

**SUPPLEMENTAL MATERIALS**

| Appendix Table 1. Sample sizes, entire sample | Page 3 |
| --- | --- |
| Appendix Table 2. Sample sizes, 55-64 years | Page 3 |
| Appendix Table 3. Sample sizes, ≥65 years | Page 3 |
| Appendix Table 4. Sample sizes, ≤ 138% Federal Poverty Level | Page 4 |
| Appendix Table 5. Sample sizes, >138% Federal Poverty Level | Page 4 |
| Appendix Figure 1. Analytic sample construction | Page 5 |
| Appendix Figure 2. Percent of people who had functional difficulties, among individuals ages 55 to 64 years, HRS 2008-2018 | Page 6 |
| Appendix Figure 3. Percent of people with unaddressed functional difficulties, among individuals ages 55 to 64 years, HRS 2008-2018 | Page 7 |
| Appendix Figure 4. Percent of people who had functional difficulties, among individuals ages 65 years or older, HRS 2008-2018 | Page 8 |
| Appendix Figure 5. Percent of people with unaddressed functional difficulties, among individuals ages 65 years or older, HRS 2008-2018 | Page 9 |
| Appendix Figure 6. Percent of people who had functional difficulties, among individuals in households below 138% FPL, HRS 2008-2018 | Page 10 |
| Appendix Figure 7. Percent of people who had functional difficulties, among individuals in households above 138% FPL, HRS 2008-2018 | Page 11 |
| Appendix Figure 8. Percent of people with unaddressed functional difficulties if assistive devices provided insufficient support, HRS 2008-2018 | Page 12 |
| Appendix Figure 9. Percent of people with functional difficulties who received any care support, HRS 2008-2018. | Page 13 |
| Appendix Figure 10. Average number of difficulties among people with functional difficulties, HRS 2008-2018. | Page 14 |
| Appendix Figure 11. Percent of people who had functional difficulties (ADLs), HRS waves 2008-2018 | Page 15 |
| Appendix Figure 12. Percent of people who had functional difficulties (IADLs), HRS waves 2008-2018 | Page 16 |
| Appendix Figure 13. Percent of people with unaddressed functional difficulties (ADLs), HRS waves 2008-2018. | Page 17 |
| Appendix Figure 14. Percent of people with unaddressed functional difficulties (IADLs), HRS waves 2008-2018. | Page 18 |
| Appendix Figure 15. Percent of people with unaddressed functional difficulties (ADLs), by household-income-to-poverty ratio, HRS waves 2008-2018. | Page 19 |
| Appendix Figure 16. Percent of people with unaddressed functional difficulties (IADLs), by household-income-to-poverty ratio, HRS waves 2008-2018. | Page 20 |

| **Appendix Table 1**. Sample sizes, entire sample | | | | | | |
| --- | --- | --- | --- | --- | --- | --- |
|  | **White** | | **Black** | | **Hispanic** | |
|  | *All* | *≥1* | *All* | *≥1* | *All* | *≥1* |
| 2008 | 11,614 | 2,894 | 2,184 | 751 | 1,018 | 367 |
| 2010 | 11,565 | 2,955 | 2,954 | 1,044 | 1,271 | 465 |
| 2012 | 11,313 | 2,864 | 3,081 | 1,076 | 1,332 | 448 |
| 2014 | 10,727 | 2,815 | 3,186 | 1,144 | 1,356 | 515 |
| 2016 | 10,096 | 2,565 | 3,406 | 1,211 | 1,429 | 492 |
| 2018 | 8,608 | 2,177 | 3,135 | 1,081 | 1,295 | 449 |
| All | 63,923 | 16,270 | 17,946 | 6,307 | 7,701 | 2,736 |
| **Notes**: *All* = all observations; *≥1* = observations with at least 1 (I)ADL needs. activities of daily living (ADL): eating, dressing, bathing, toileting, walking and getting in/out of bed. Instrumental activities of daily living (IADL): meal preparation, grocery shopping, making phone calls, managing money. | | | | | | |

| **Appendix Table 2**. Sample sizes, 55-64 years | | | | | | |
| --- | --- | --- | --- | --- | --- | --- |
|  | **White** | | **Black** | | **Hispanic** | |
|  | *All* | *≥1* | *All* | *≥1* | *All* | *≥1* |
| 2008 | 3,279 | 477 | 714 | 182 | 306 | 82 |
| 2010 | 3,659 | 590 | 1,494 | 457 | 567 | 157 |
| 2012 | 3,601 | 572 | 1,665 | 482 | 617 | 157 |
| 2014 | 3,435 | 575 | 1,739 | 523 | 642 | 194 |
| 2016 | 3,436 | 594 | 1,836 | 556 | 680 | 154 |
| 2018 | 2,800 | 501 | 1,600 | 486 | 558 | 126 |
| All | 20,210 | 3,309 | 9,048 | 2,686 | 3,370 | 870 |
| **Notes**: *All* = all observations; *≥1* = observations with at least 1 (I)ADL needs. activities of daily living (ADL): eating, dressing, bathing, toileting, walking and getting in/out of bed. Instrumental activities of daily living (IADL): meal preparation, grocery shopping, making phone calls, managing money, managing medications. | | | | | | |

| **Appendix Table 3**. Sample sizes, ≥65 years | | | | | | |
| --- | --- | --- | --- | --- | --- | --- |
|  | **White** | | **Black** | | **Hispanic** | |
|  | *All* | *≥1* | *All* | *≥1* | *All* | *≥1* |
| 2008 | 8,335 | 2,417 | 1,470 | 569 | 712 | 285 |
| 2010 | 7,906 | 2,365 | 1,460 | 587 | 704 | 308 |
| 2012 | 7,712 | 2,292 | 1,416 | 594 | 715 | 291 |
| 2014 | 7,292 | 2,240 | 1,447 | 621 | 714 | 321 |
| 2016 | 6,660 | 1,971 | 1,570 | 655 | 749 | 338 |
| 2018 | 5,808 | 1,676 | 1,535 | 595 | 737 | 323 |
| All | 43,713 | 12,961 | 8,898 | 3,621 | 4,331 | 1,866 |
| **Notes**: *All* = all observations; *≥1* = observations with at least 1 (I)ADL needs. activities of daily living (ADL): eating, dressing, bathing, toileting, walking and getting in/out of bed. Instrumental activities of daily living (IADL): meal preparation, grocery shopping, making phone calls, managing money, managing medications. | | | | | | |

| **Appendix Table 4**. Sample sizes, ≤ 138% Federal Poverty Level | | | | | | |
| --- | --- | --- | --- | --- | --- | --- |
|  | **White** | | **Black** | | **Hispanic** | |
|  | *All* | *≥1* | *All* | *≥1* | *All* | *≥1* |
| 2008 | 1,154 | 512 | 696 | 339 | 373 | 174 |
| 2010 | 1,315 | 561 | 1,052 | 506 | 521 | 248 |
| 2012 | 1,329 | 579 | 1,114 | 533 | 555 | 225 |
| 2014 | 1,208 | 567 | 1,165 | 575 | 560 | 268 |
| 2016 | 1,171 | 517 | 1,176 | 588 | 585 | 265 |
| 2018 | 1,039 | 458 | 1,094 | 536 | 512 | 217 |
| All | 7,216 | 3,194 | 6,297 | 3,077 | 3,106 | 1,397 |
| **Notes**: *All* = all observations; *≥1* = observations with at least 1 (I)ADL needs. activities of daily living (ADL): eating, dressing, bathing, toileting, walking and getting in/out of bed. Instrumental activities of daily living (IADL): meal preparation, grocery shopping, making phone calls, managing money, managing medications. | | | | | | |

| **Appendix Table 5**. Sample sizes, >138% Federal Poverty Level | | | | | | |
| --- | --- | --- | --- | --- | --- | --- |
|  | **White** | | **Black** | | **Hispanic** | |
|  | *All* | *≥1* | *All* | *≥1* | *All* | *≥1* |
| 2008 | 10,460 | 2,382 | 1,488 | 412 | 645 | 193 |
| 2010 | 10,250 | 2,394 | 1,902 | 538 | 750 | 217 |
| 2012 | 9,984 | 2,285 | 1,967 | 543 | 777 | 223 |
| 2014 | 9,519 | 2,248 | 2,021 | 569 | 796 | 247 |
| 2016 | 8,925 | 2,048 | 2,230 | 623 | 844 | 227 |
| 2018 | 7,569 | 1,719 | 2,041 | 545 | 783 | 232 |
| All | 56,707 | 13,076 | 11,649 | 3,230 | 4,595 | 1,339 |
| **Notes**: *All* = all observations; *≥1* = observations with at least 1 (I)ADL needs. activities of daily living (ADL): eating, dressing, bathing, toileting, walking and getting in/out of bed. Instrumental activities of daily living (IADL): meal preparation, grocery shopping, making phone calls, managing money, managing medications. | | | | | | |

**Appendix Figure 1**. Analytic sample construction.

**Appendix Figure 2.** Percent of people who had functional difficulties, among individuals ages 55 to 64 years, HRS 2008-2018.


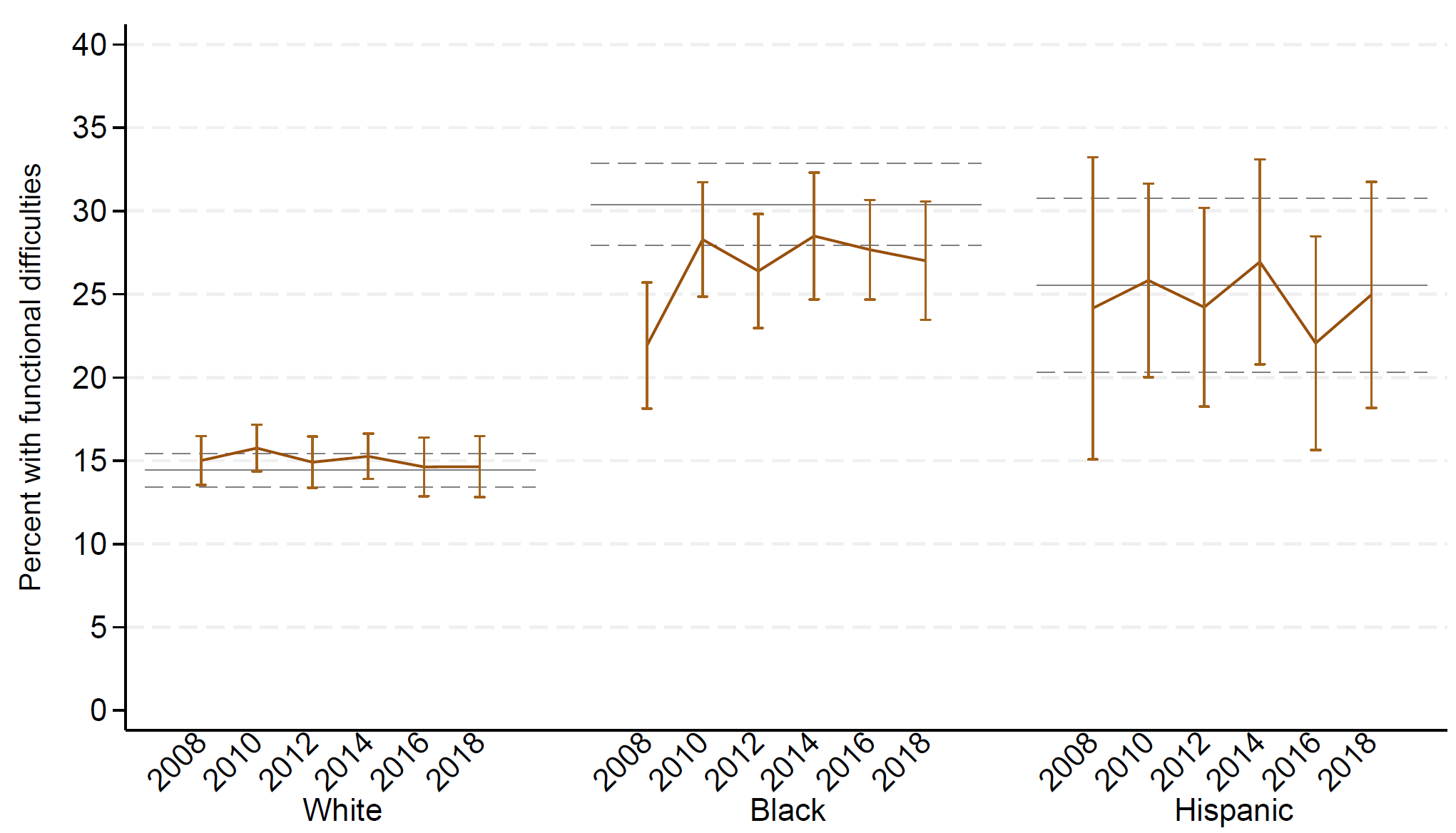


**Source**: Authors’ calculations using data from the 2008-2018 Health and Retirement Study (HRS) for community-dwelling individuals at least 55 years of age.

**Notes**: All statistics were weighted to account for sampling and to make nationally representative. Annual estimates and associated 95% confidence intervals were derived from HRS cross-sections and adjusted for sex, age, marital status, and children. Average across all waves and associated 95% confidence intervals were derived from pooled cross-sections. ADL = Activities of daily living included eating, dressing, bathing, walking, getting into or out of bed, and using the toilet. IADL = Instrumental activities of daily living included meal preparation, grocery shopping, making phone calls, managing money, and managing medications. Functional difficulty were respondents that reported (or a proxy reported them as) having difficulty with the activity due to health or memory problems.

**Appendix Figure 3.** Percent of people with unaddressed functional difficulties, among individuals ages 55 to 64 years, HRS 2008-2018.


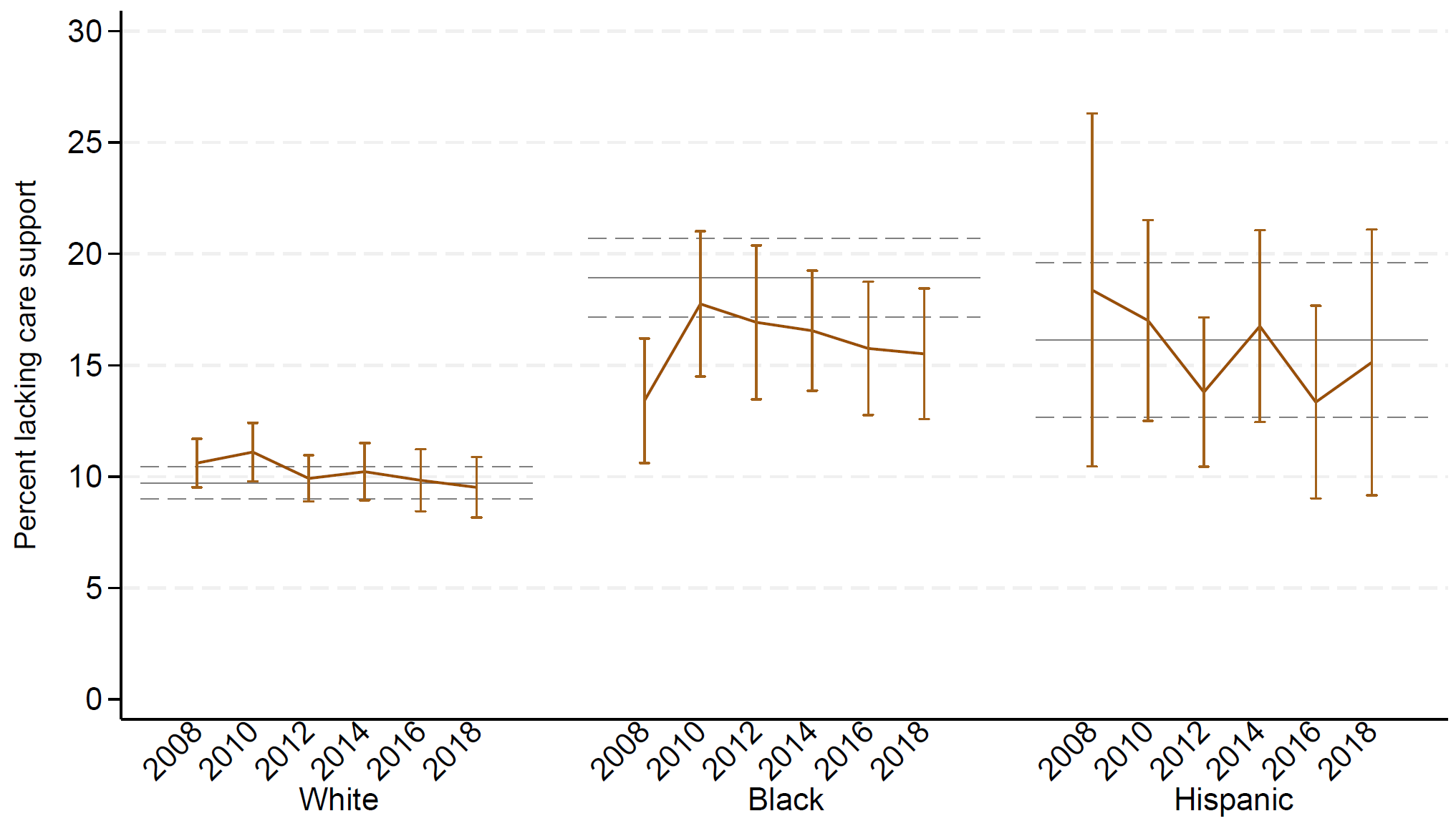


**Source**: Authors’ calculations using data from the 2008-2018 Health and Retirement Study (HRS) for community-dwelling individuals at least 55 years of age.

**Notes**: All statistics were weighted to account for sampling and to make nationally representative. Annual estimates and associated 95% confidence intervals were derived from HRS cross-sections and adjusted for sex, age, marital status, and children. Average across all waves and associated 95% confidence intervals were derived from pooled cross-sections. ADL = Activities of daily living included eating, dressing, bathing, walking, getting into or out of bed, and using the toilet. IADL = Instrumental activities of daily living included meal preparation, grocery shopping, making phone calls, managing money, and managing medications. People lacking care support referred to respondents that reported (or a proxy reported them as) having difficulty with the activity due to health or memory problems but did not receive assistance from a family or formal caregiver or through the use of relevant equipment.

**Appendix Figure 4.** Percent of people who had functional difficulties, among individuals ages 65 years or older, HRS 2008-2018.


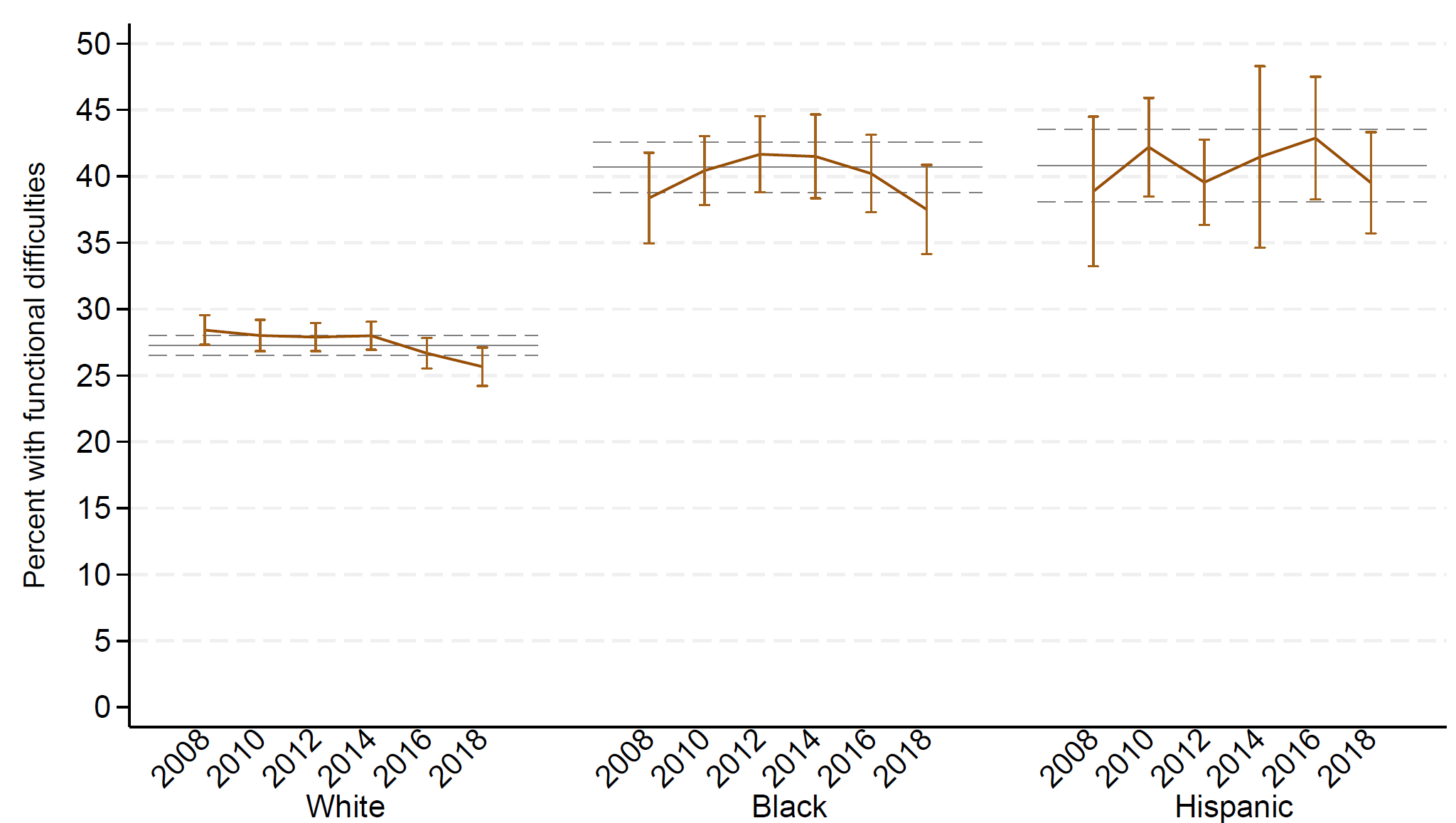


**Source**: Authors’ calculations using data from the 2008-2018 Health and Retirement Study (HRS) for community-dwelling individuals at least 55 years of age.

**Notes**: All statistics were weighted to account for sampling and to make nationally representative. Annual estimates and associated 95% confidence intervals were derived from HRS cross-sections and adjusted for sex, age, marital status, and children. Average across all waves and associated 95% confidence intervals were derived from pooled cross-sections. ADL = Activities of daily living included eating, dressing, bathing, walking, getting into or out of bed, and using the toilet. IADL = Instrumental activities of daily living included meal preparation, grocery shopping, making phone calls, managing money, and managing medications. Functional difficulty were respondents that reported (or a proxy reported them as) having difficulty with the activity due to health or memory problems.

**Appendix Figure 5.** Percent of people with unaddressed functional difficulties, among individuals ages 65 years or older, HRS 2008-2018.


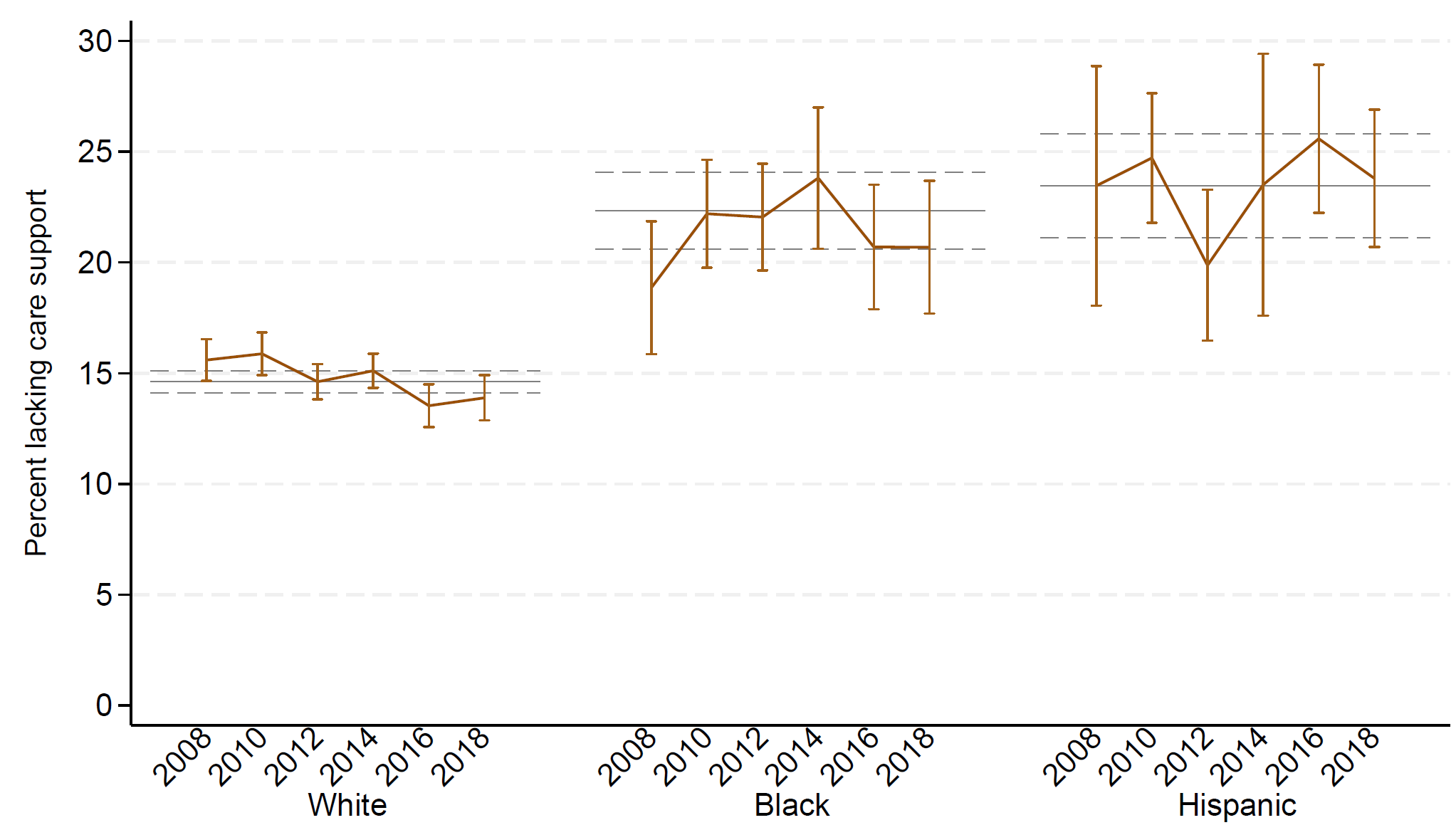


**Source**: Authors’ calculations using data from the 2008-2018 Health and Retirement Study (HRS) for community-dwelling individuals at least 55 years of age.

**Notes**: All statistics were weighted to account for sampling and to make nationally representative. Annual estimates and associated 95% confidence intervals were derived from HRS cross-sections and adjusted for sex, age, marital status, and children. Average across all waves and associated 95% confidence intervals were derived from pooled cross-sections. ADL = Activities of daily living included eating, dressing, bathing, walking, getting into or out of bed, and using the toilet. IADL = Instrumental activities of daily living included meal preparation, grocery shopping, making phone calls, managing money, and managing medications. People lacking care support referred to respondents that reported (or a proxy reported them as) having difficulty with the activity due to health or memory problems but did not receive assistance from a family or formal caregiver or through the use of relevant equipment.

**Appendix Figure 6.** Percent of people who had functional difficulties, among individuals in households below 138% FPL, HRS 2008-2018.


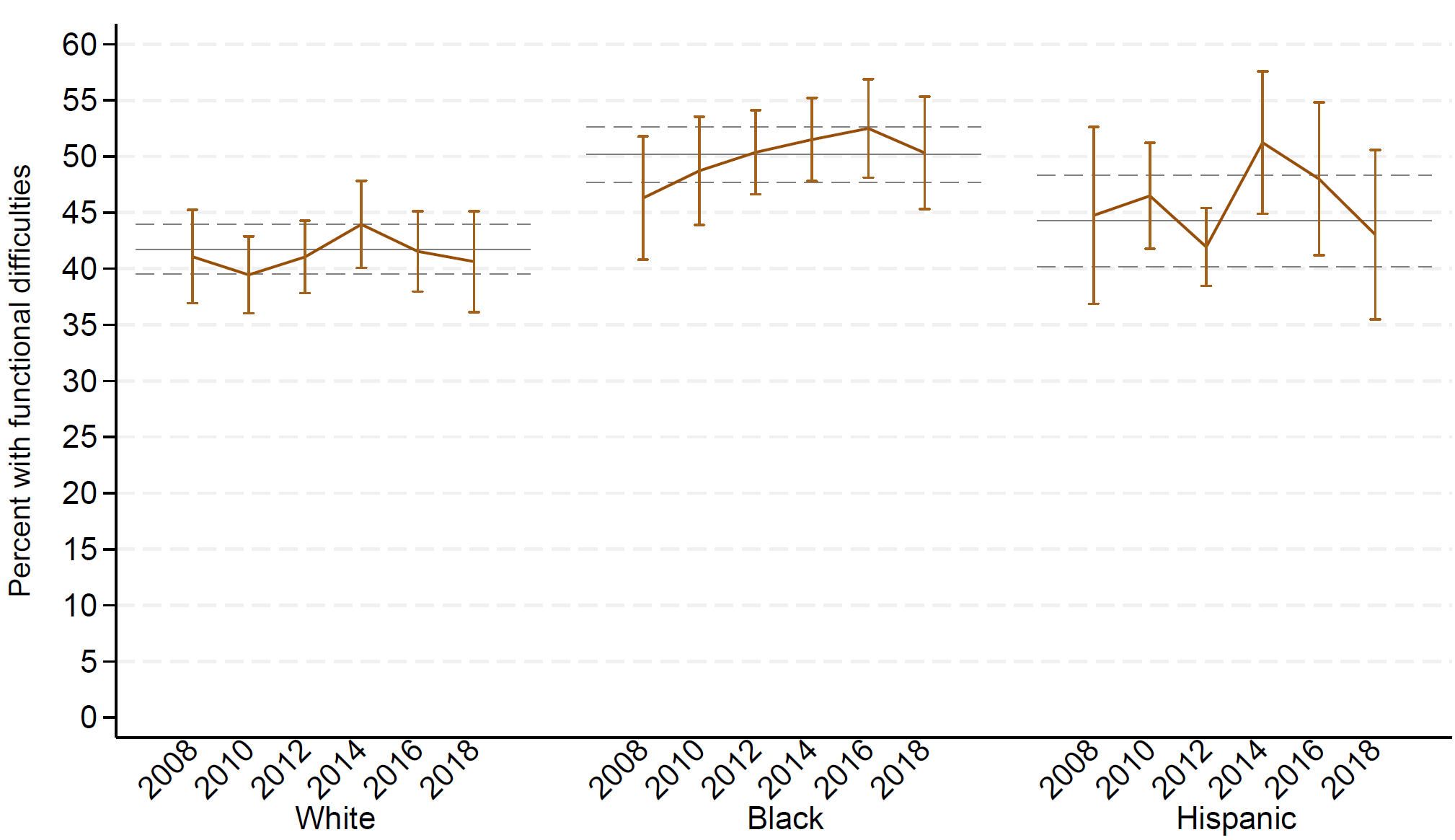


**Source**: Authors’ calculations using data from the 2008-2018 Health and Retirement Study (HRS) for community-dwelling individuals at least 55 years of age.

**Notes**: All statistics were weighted to account for sampling and to make nationally representative. Annual estimates and associated 95% confidence intervals were derived from HRS cross-sections and adjusted for sex, age, marital status, and children. Average across all waves and associated 95% confidence intervals were derived from pooled cross-sections. ADL = Activities of daily living included eating, dressing, bathing, walking, getting into or out of bed, and using the toilet. IADL = Instrumental activities of daily living included meal preparation, grocery shopping, making phone calls, managing money, and managing medications. Functional difficulty were respondents that reported (or a proxy reported them as) having difficulty with the activity due to health or memory problems.

**Appendix Figure 7.** Percent of people who had functional difficulties, among individuals in households above 138% FPL, HRS 2008-2018.


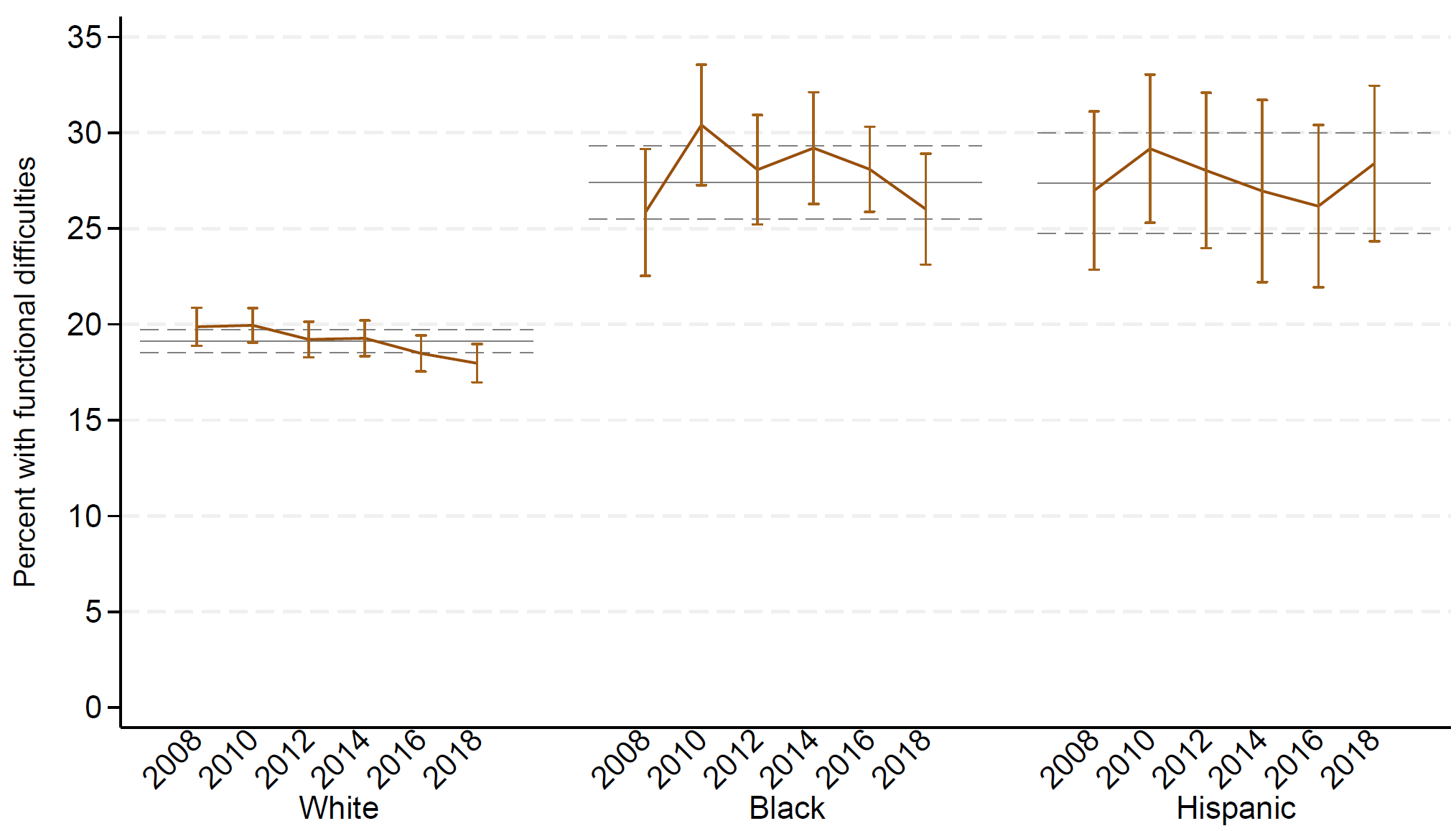


**Source**: Authors’ calculations using data from the 2008-2018 Health and Retirement Study (HRS) for community-dwelling individuals at least 55 years of age.

**Notes**: All statistics were weighted to account for sampling and to make nationally representative and adjusted for sex, age, marital status, and children. Annual estimates and associated 95% confidence intervals were derived from HRS cross-sections. Average across all waves and associated 95% confidence intervals were derived from pooled cross-sections. ADL = Activities of daily living included eating, dressing, bathing, walking, getting into or out of bed, and using the toilet. IADL = Instrumental activities of daily living included meal preparation, grocery shopping, making phone calls, managing money, and managing medications. Functional difficulty were respondents that reported (or a proxy reported them as) having difficulty with the activity due to health or memory problems.

**Appendix Figure 8.** Percent of people with unaddressed functional difficulties if assistive devices provided insufficient support, HRS 2008-2018.


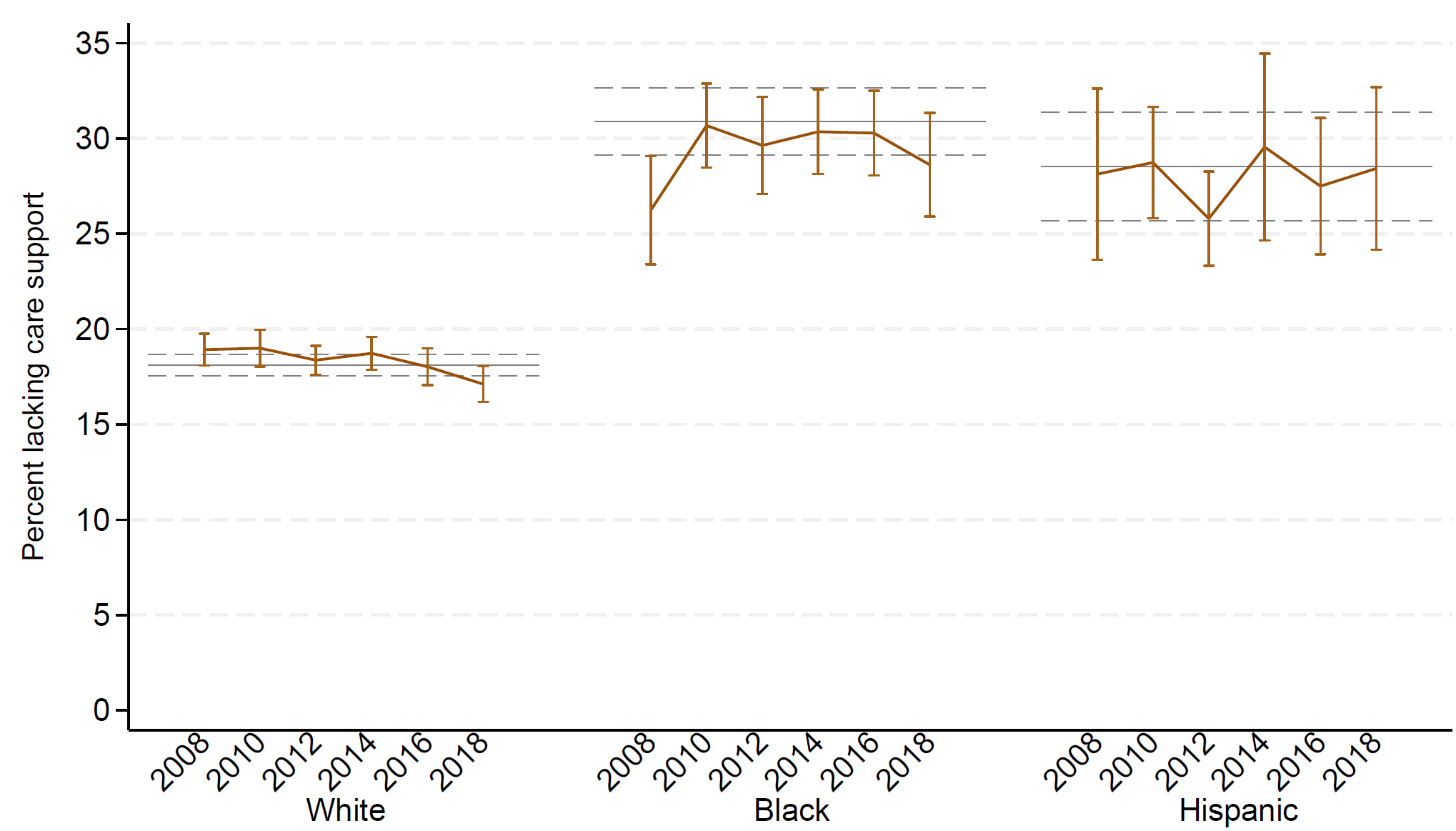


**Source**: Authors’ calculations using data from the 2008-2018 Health and Retirement Study (HRS) for community-dwelling individuals at least 55 years of age.

**Notes**: All statistics were weighted to account for sampling and to make nationally representative and adjusted for sex, age, marital status, and children. Annual estimates and associated 95% confidence intervals were derived from HRS cross-sections. Average across all waves and associated 95% confidence intervals were derived from pooled cross-sections. ADL = Activities of daily living included eating, dressing, bathing, walking, getting into or out of bed, and using the toilet. IADL = Instrumental activities of daily living included meal preparation, grocery shopping, making phone calls, managing money, and managing medications. People lacking care support referred to respondents that reported (or a proxy reported them as) having difficulty with the activity due to health or memory problems but did not receive assistance from a family or formal caregiver or through the use of relevant equipment.

**Appendix Figure 9.** Percent of people with functional difficulties who received any care support, HRS 2008-2018.


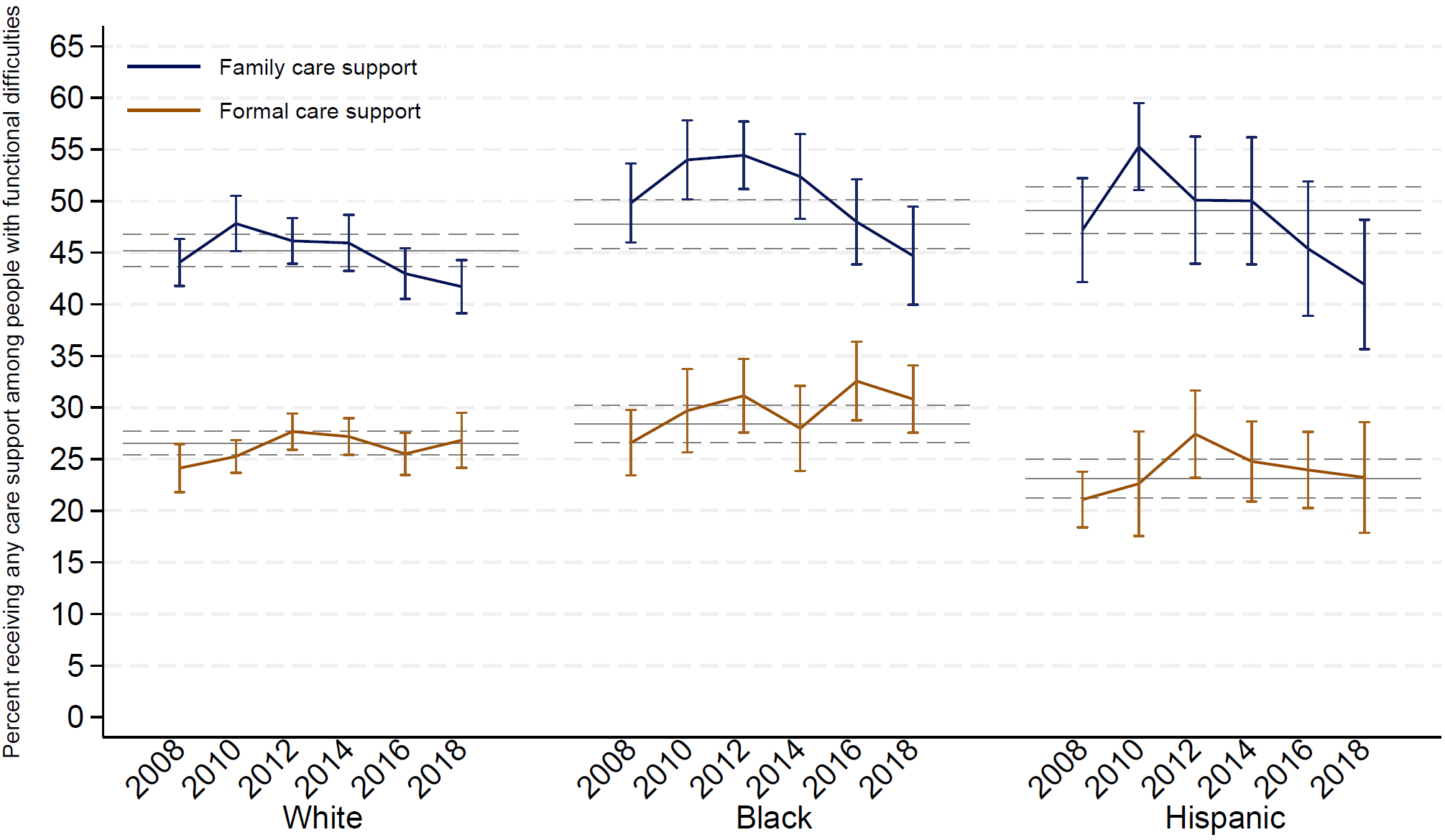


**Source**: Authors’ calculations using data from the 2008-2018 Health and Retirement Study (HRS) for community-dwelling individuals at least 55 years of age.

**Notes**: All statistics were weighted to account for sampling and to make nationally representative. Annual estimates and associated 95% confidence intervals were derived from HRS cross-sections and adjusted for sex, age, marital status, and children. Average across all waves and associated 95% confidence intervals were derived from pooled cross-sections. Informal care hours included hours of uncompensated care from family (e.g., spouse/partner) and friends. Formal care hours included care received from an organization, an “institution” employee, a paid helper, or a health care professional.

**Appendix Figure 10.** Average number of difficulties among people with functional difficulties, HRS 2008-2018.


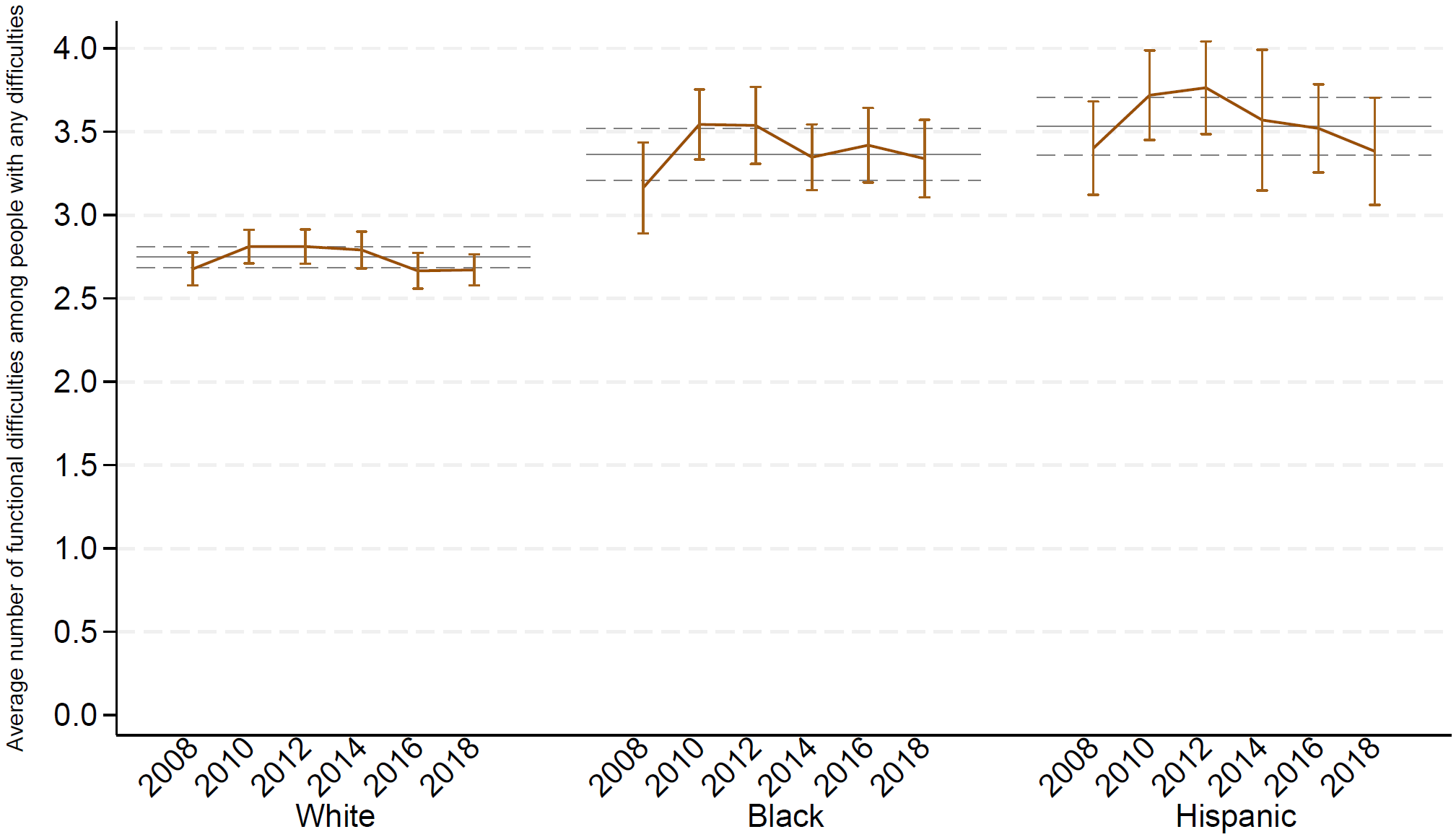


**Source**: Authors’ calculations using data from the 2008-2018 Health and Retirement Study (HRS) for community-dwelling individuals at least 55 years of age.

**Notes**: All statistics were weighted to account for sampling and to make nationally representative. Annual estimates and associated 95% confidence intervals were derived from HRS cross-sections and adjusted for sex, age, marital status, and children. Average across all waves and associated 95% confidence intervals were derived from pooled cross-sections. ADL = Activities of daily living included eating, dressing, bathing, walking, getting into or out of bed, and using the toilet. IADL = Instrumental activities of daily living included meal preparation, grocery shopping, making phone calls, managing money, and managing medications.

**Appendix Figure 11.** Percent of people who had functional difficulties (ADLs), HRS waves 2008-2018


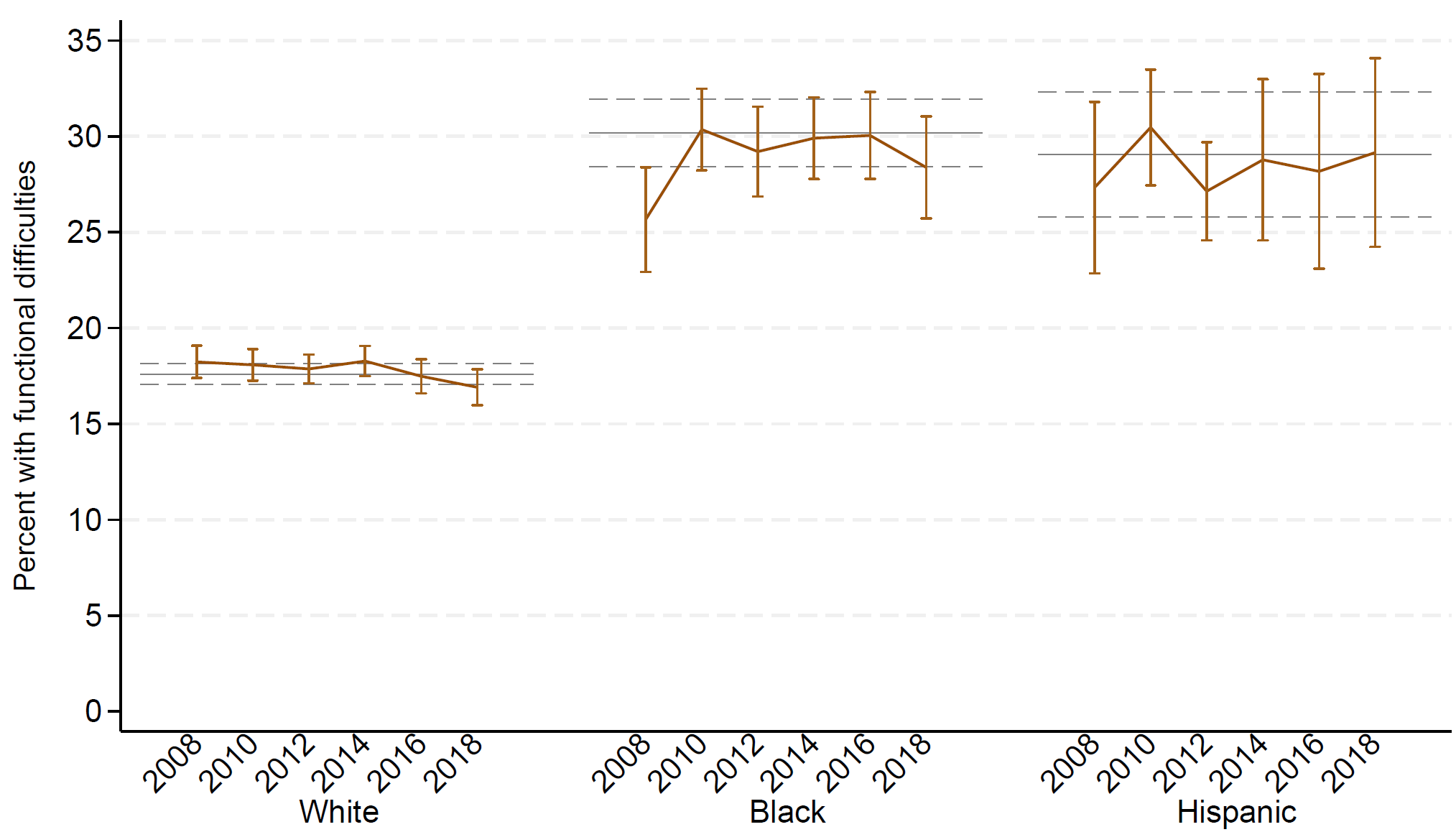


**Source**: Authors’ calculations using data from the 2008-2018 Health and Retirement Study (HRS) for community-dwelling individuals at least 55 years of age.

**Notes**: All statistics were weighted to account for sampling and to make nationally representative. Annual estimates and associated 95% confidence intervals were derived from HRS cross-sections and adjusted for sex, age, marital status, and children. Average across all waves and associated 95% confidence intervals were derived from pooled cross-sections. ADL = Activities of daily living included eating, dressing, bathing, walking, getting into or out of bed, and using the toilet. IADL = Instrumental activities of daily living included meal preparation, grocery shopping, making phone calls, managing money, and managing medications. People lacking care support referred to respondents that reported (or a proxy reported them as) having difficulty with the activity due to health or memory problems but did not receive assistance from a family or formal caregiver or through the use of relevant equipment.

**Appendix Figure 12.** Percent of people who had functional difficulties (IADLs), HRS waves 2008-2018


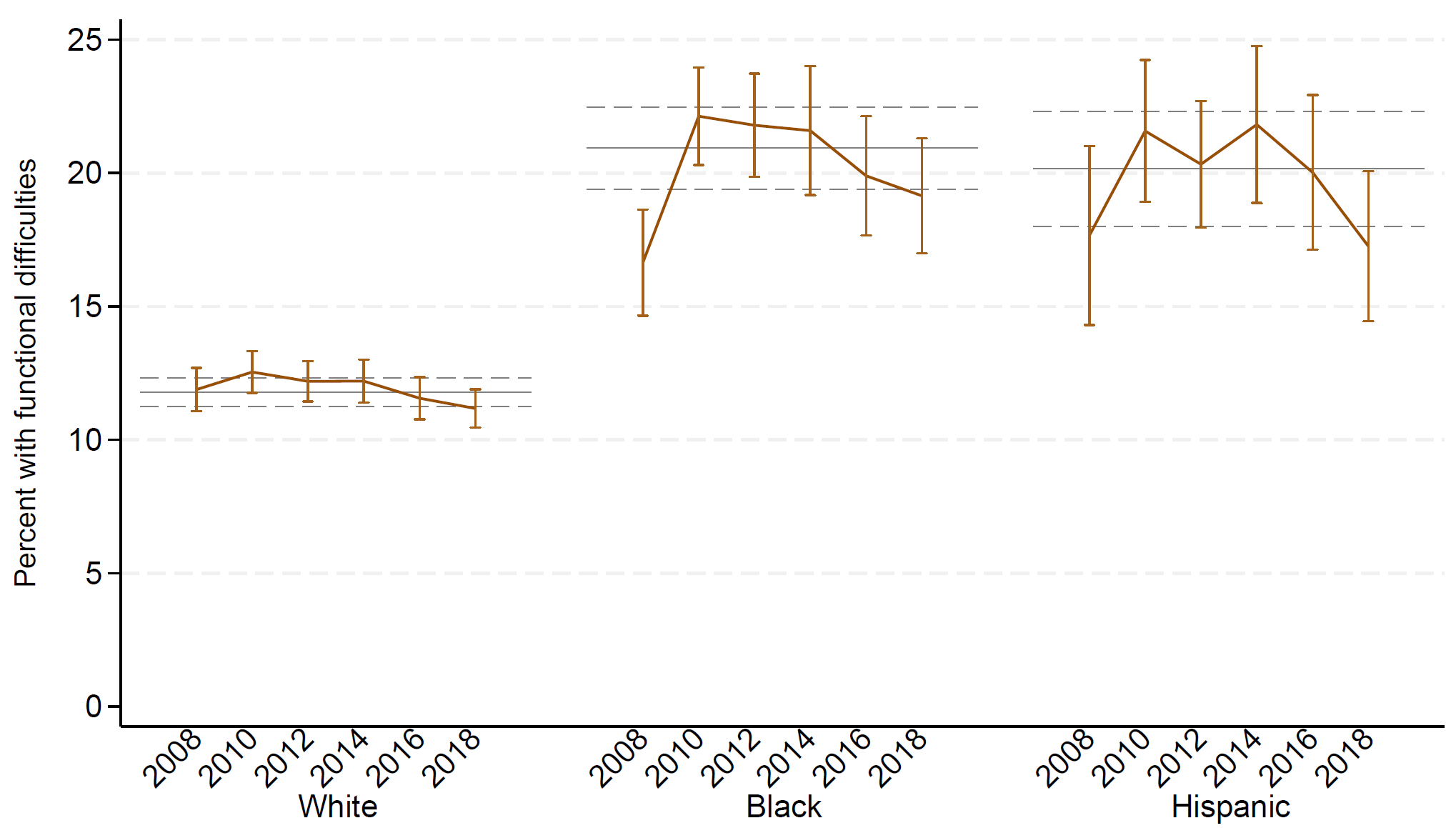


**Source**: Authors’ calculations using data from the 2008-2018 Health and Retirement Study (HRS) for community-dwelling individuals at least 55 years of age.

**Notes**: All statistics were weighted to account for sampling and to make nationally representative. Annual estimates and associated 95% confidence intervals were derived from HRS cross-sections and adjusted for sex, age, marital status, and children. Average across all waves and associated 95% confidence intervals were derived from pooled cross-sections. ADL = Activities of daily living included eating, dressing, bathing, walking, getting into or out of bed, and using the toilet. IADL = Instrumental activities of daily living included meal preparation, grocery shopping, making phone calls, managing money, and managing medications. People lacking care support referred to respondents that reported (or a proxy reported them as) having difficulty with the activity due to health or memory problems but did not receive assistance from a family or formal caregiver or through the use of relevant equipment.

**Appendix Figure 13.** Percent of people with unaddressed functional difficulties (ADLs), HRS waves 2008-2018.


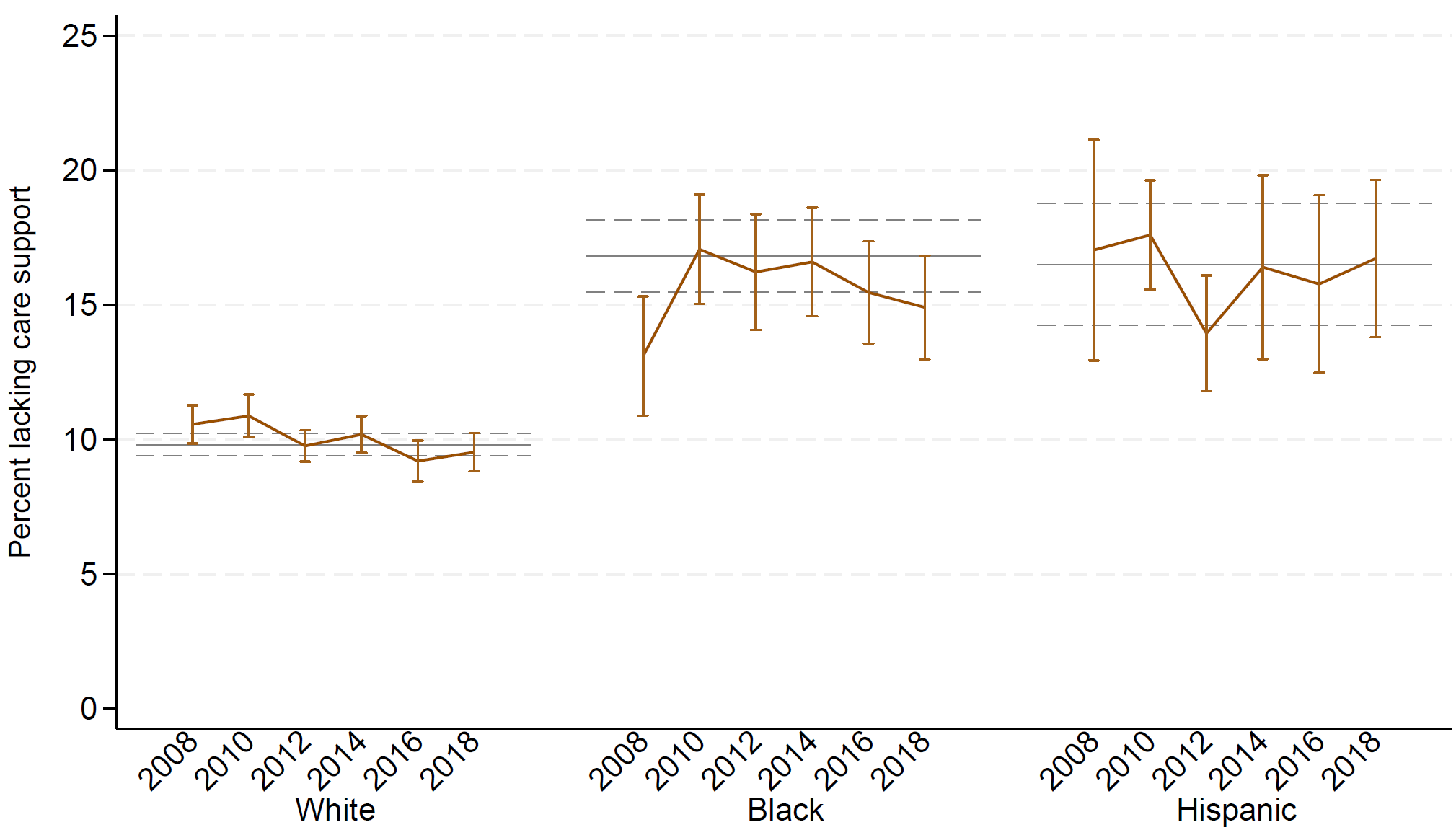


**Source**: Authors’ calculations using data from the 2008-2018 Health and Retirement Study (HRS) for community-dwelling individuals at least 55 years of age.

**Notes**: All statistics were weighted to account for sampling and to make nationally representative. Annual estimates and associated 95% confidence intervals were derived from HRS cross-sections and adjusted for sex, age, marital status, and children. Average across all waves and associated 95% confidence intervals were derived from pooled cross-sections. ADL = Activities of daily living included eating, dressing, bathing, walking, getting into or out of bed, and using the toilet. IADL = Instrumental activities of daily living included meal preparation, grocery shopping, making phone calls, managing money, and managing medications. People lacking care support referred to respondents that reported (or a proxy reported them as) having difficulty with the activity due to health or memory problems but did not receive assistance from a family or formal caregiver or through the use of relevant equipment.

**Appendix Figure 14.** Percent of people with unaddressed functional difficulties (IADLs), HRS waves 2008-2018.


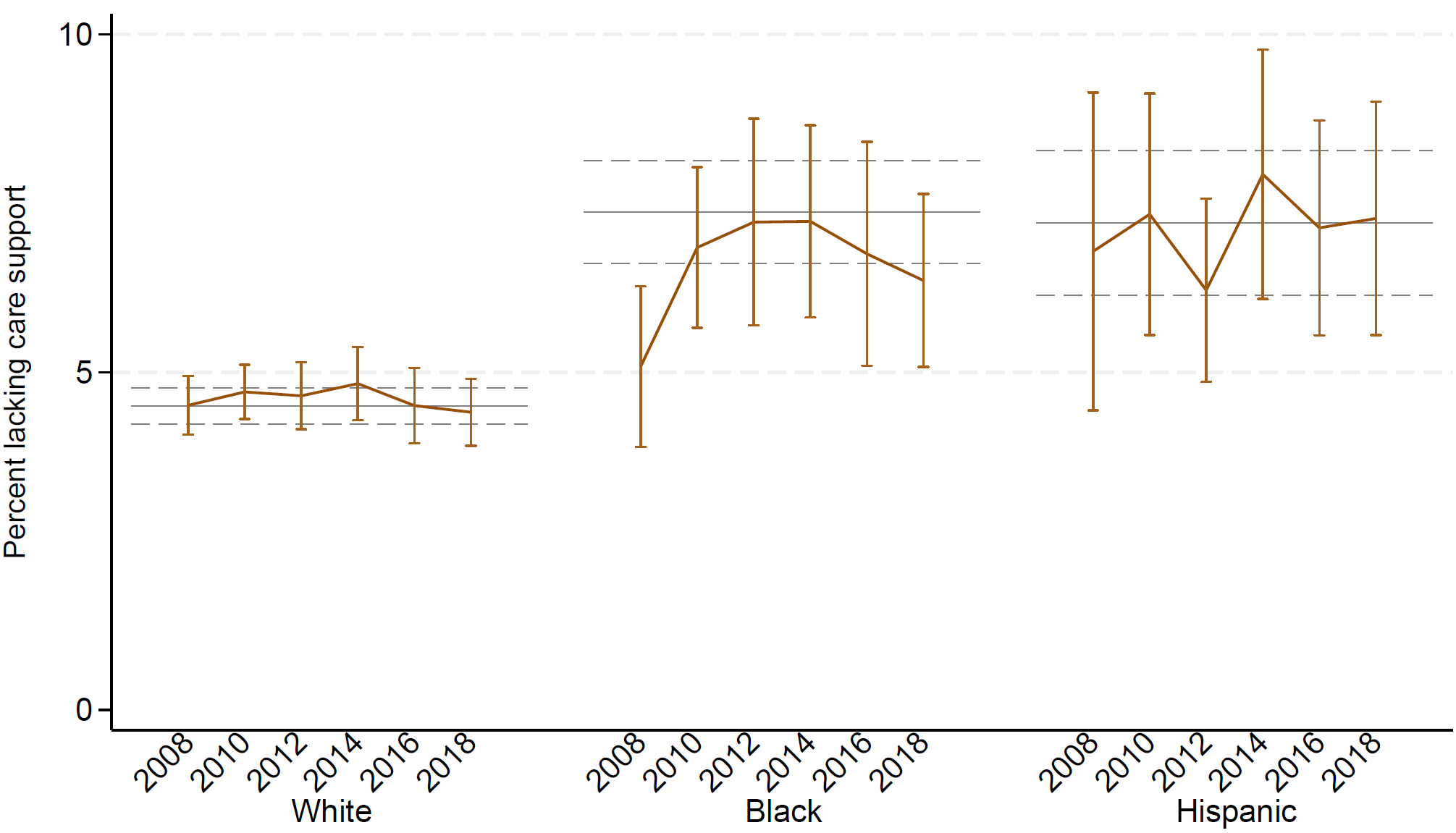


**Source**: Authors’ calculations using data from the 2008-2018 Health and Retirement Study (HRS) for community-dwelling individuals at least 55 years of age.

**Notes**: All statistics were weighted to account for sampling and to make nationally representative. Annual estimates and associated 95% confidence intervals were derived from HRS cross-sections and adjusted for sex, age, marital status, and children. Average across all waves and associated 95% confidence intervals were derived from pooled cross-sections. ADL = Activities of daily living included eating, dressing, bathing, walking, getting into or out of bed, and using the toilet. IADL = Instrumental activities of daily living included meal preparation, grocery shopping, making phone calls, managing money, and managing medications. People lacking care support referred to respondents that reported (or a proxy reported them as) having difficulty with the activity due to health or memory problems but did not receive assistance from a family or formal caregiver or through the use of relevant equipment.

**Appendix Figure 15.** Percent of people with unaddressed functional difficulties (ADLs), by household-income-to-poverty ratio, HRS waves 2008-2018.


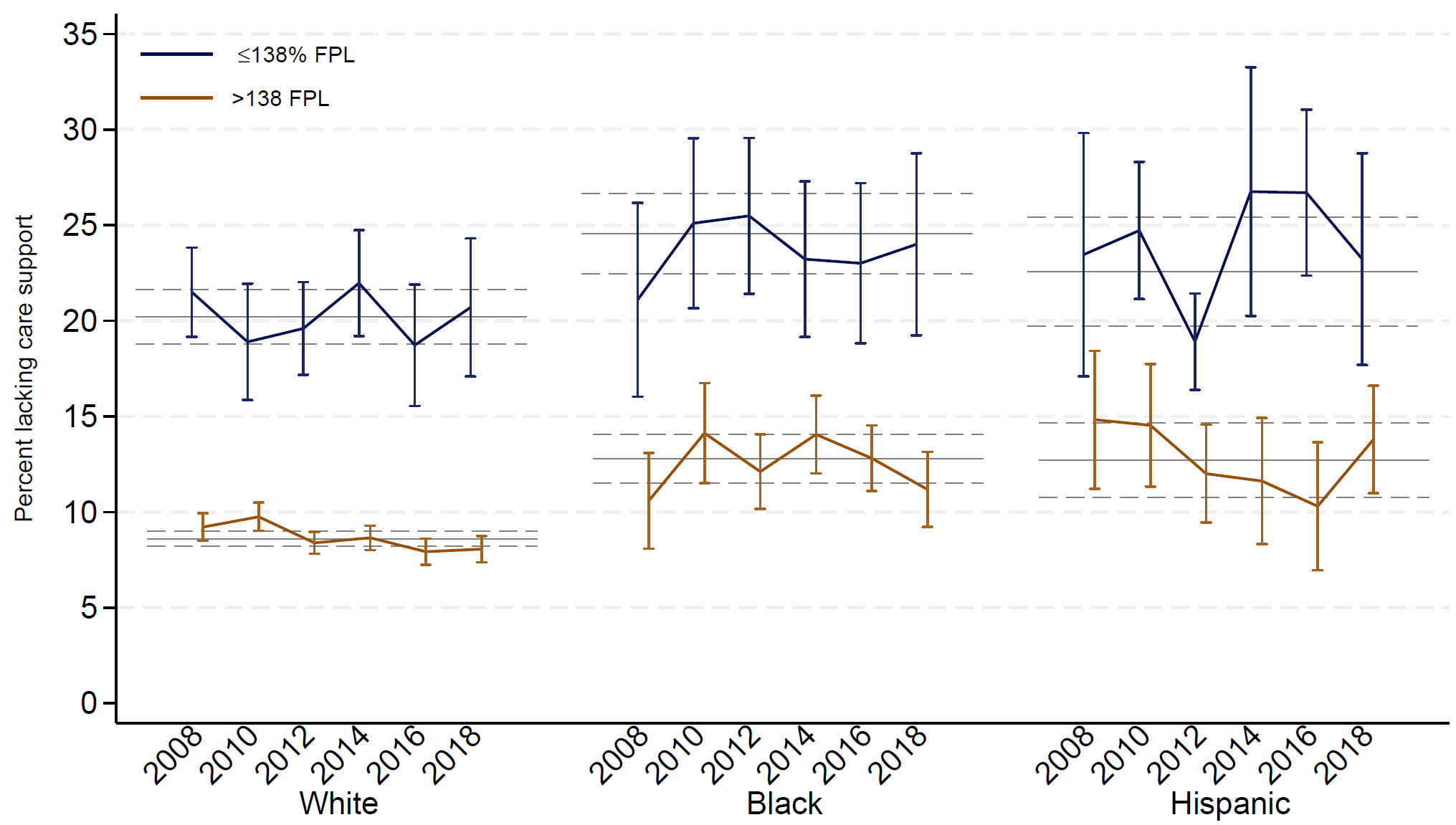


**Source**: Authors’ calculations using data from the 2008-2018 Health and Retirement Study (HRS) for community-dwelling individuals at least 55 years of age.

**Notes**: All statistics were weighted to account for sampling and to make nationally representative. Annual estimates and associated 95% confidence intervals were derived from HRS cross-sections and adjusted for sex, age, marital status, and children. Average across all waves and associated 95% confidence intervals were derived from pooled cross-sections. ADL = Activities of daily living included eating, dressing, bathing, walking, getting into or out of bed, and using the toilet. IADL = Instrumental activities of daily living included meal preparation, grocery shopping, making phone calls, managing money, and managing medications. People lacking care support referred to respondents that reported (or a proxy reported them as) having difficulty with the activity due to health or memory problems but did not receive assistance from a family or formal caregiver or through the use of relevant equipment.

**Appendix Figure 16.** Percent of people with unaddressed functional difficulties (IADLs), by household-income-to-poverty ratio, HRS waves 2008-2018.


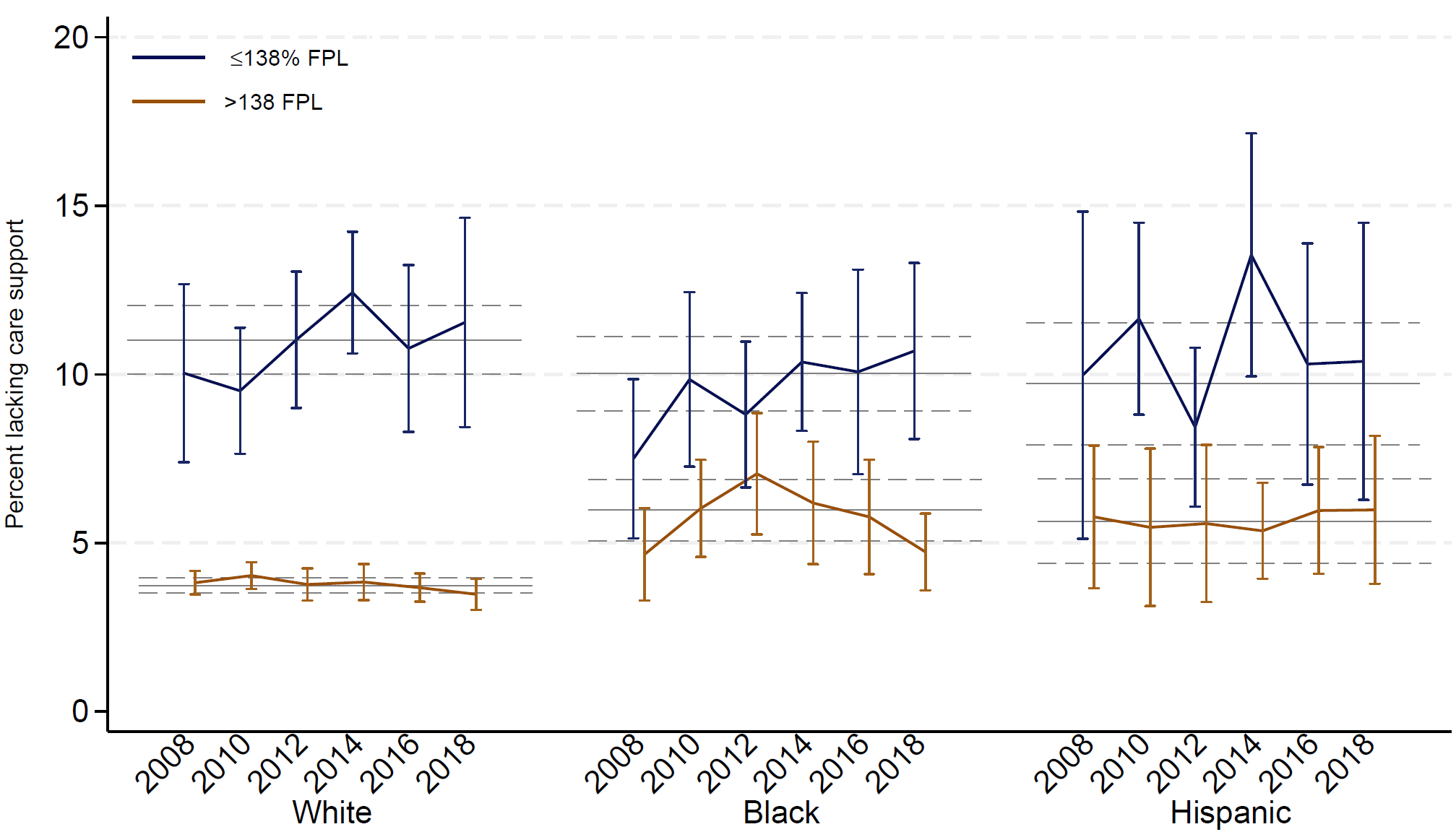


**Source**: Authors’ calculations using data from the 2008-2018 Health and Retirement Study (HRS) for community-dwelling individuals at least 55 years of age.

**Notes**: All statistics were weighted to account for sampling and to make nationally representative. Annual estimates and associated 95% confidence intervals were derived from HRS cross-sections and adjusted for sex, age, marital status, and children. Average across all waves and associated 95% confidence intervals were derived from pooled cross-sections. ADL = Activities of daily living included eating, dressing, bathing, walking, getting into or out of bed, and using the toilet. IADL = Instrumental activities of daily living included meal preparation, grocery shopping, making phone calls, managing money, and managing medications. People lacking care support referred to respondents that reported (or a proxy reported them as) having difficulty with the activity due to health or memory problems but did not receive assistance from a family or formal caregiver or through the use of relevant equipment.
